# Supplementary material for: Production of germ-free mosquitoes via transient colonisation allows stage-specific investigation of host–microbiota interactions
Source: Nat Commun. 2021 Feb 11;12:942. doi: 10.1038/s41467-021-21195-3 (PMC7878806; doi:10.1038/s41467-021-21195-3)
Supplement: Supplementary file 1 — Supplementary information [file 41467_2021_21195_MOESM1_ESM.pdf]

Supplementary Information

Production of germ-free mosquitoes via transient colonisation allows stage-specific investigation of host-microbiota interactions

Ottavia Romoli[1,*], Johan Claes Schönbeck[1], Siegfried Hapfelmeier[2], and Mathilde Gendrin[1, 3,*]

[1] Microbiota of Insect Vectors Group, Institut Pasteur de la Guyane, Cayenne, French Guiana, France
[2] Institute for Infectious Diseases, University of Bern, Bern, Switzerland
[3] Parasites and Insect Vectors Department, Institut Pasteur, Paris, France

* Correspondence: mathilde.gendrin@pasteur.fr; oromoli@pasteur-cayenne.fr

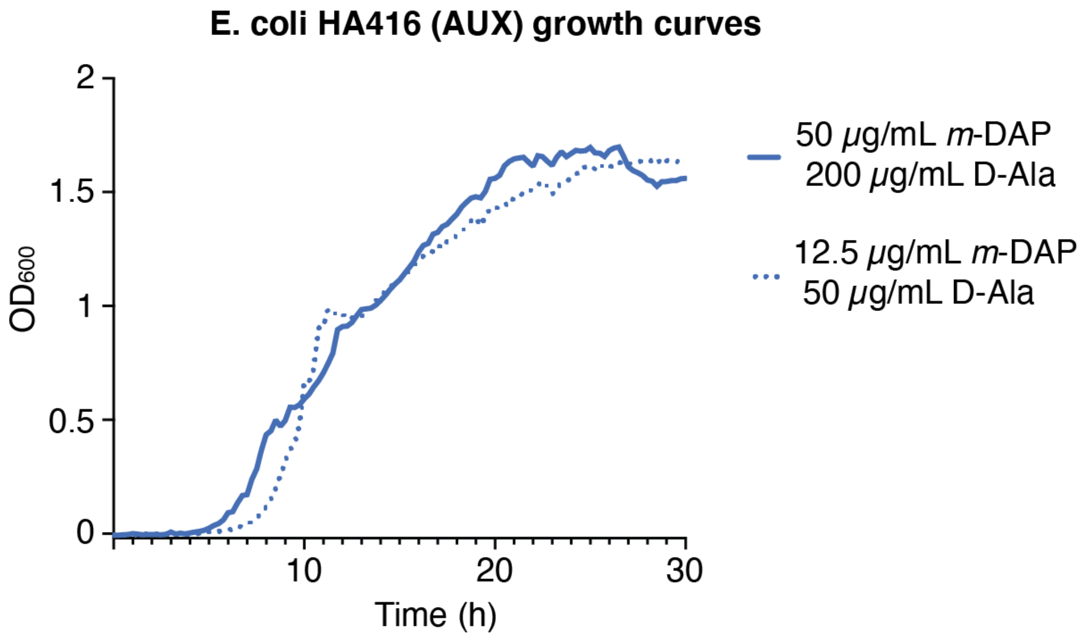

Figure S1. Growth curves of auxotrophic (AUX) *E. coli* HA416 in LB supplemented with optimal concentrations of *meso*-diaminopimelic acid (*m*-DAP) and D-alanine (D-Ala) from [1] (continuous line) and concentrations compatible with larval growth (dotted line). The experiment was performed twice with independent bacterial cultures. Source Data are provided as a Source Data file.

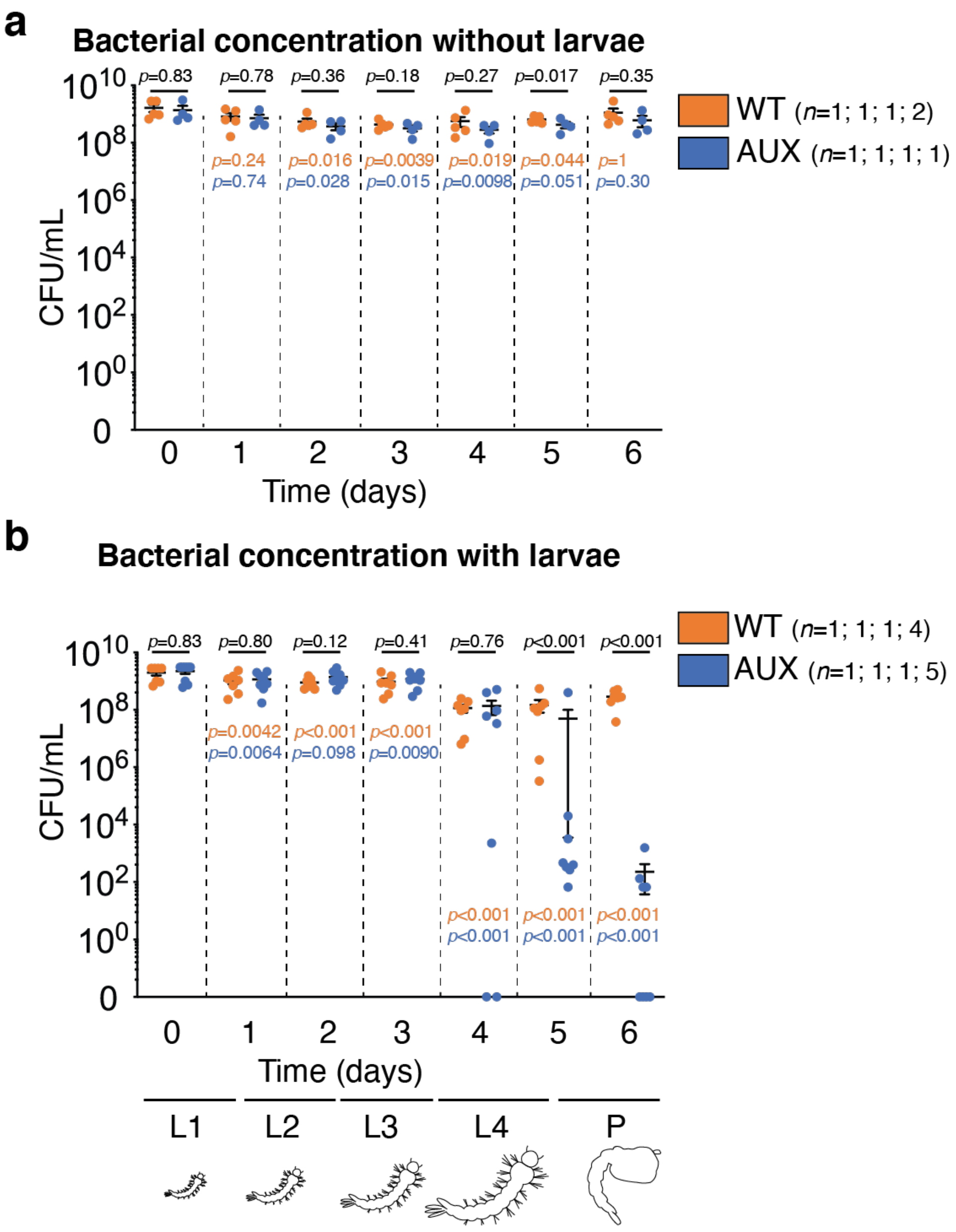

Figure S2. Bacterial concentration in larval rearing water of wild-type (WT, orange) and auxotrophic (AUX, blue) *E. coli* in absence (**a**) and presence of larvae (**b**). Data show mean ± SEM of four independent replicates. The exact number of individuals analysed per condition and replicate is indicated in each panel. Statistical significance was determined with generalized linear mixed models and least square means with Bonferroni correction. Exact *p* values are indicated in the figure: *p* values in black represent the level of significance of test comparing WT and AUX loads at each time-point; coloured *p* values represent the level of significance comparing starting loads (Day 0) with loads measured at each time point for WT (orange) and AUX (blue). See Table S1 for detailed statistical information. Source Data are provided as a Source Data file.

Bacterial load and prevalence of AUX *E. coli*

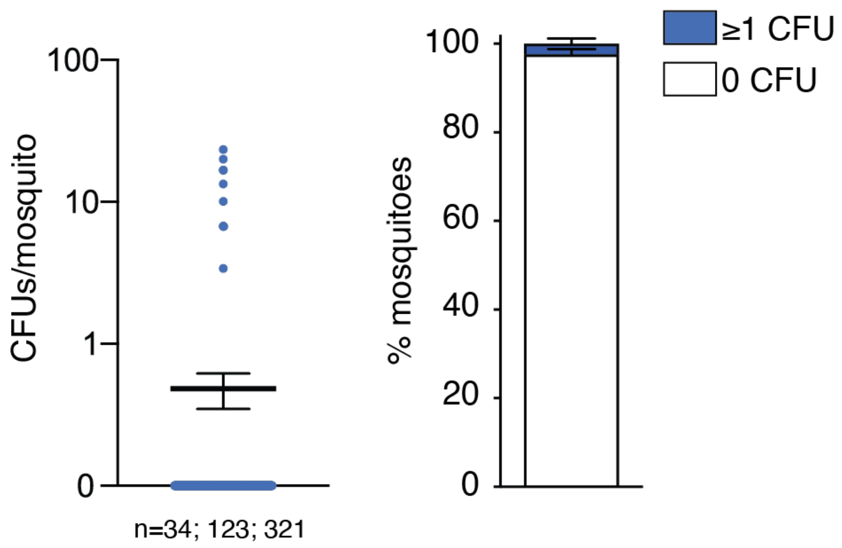

Figure S3. Bacterial load and prevalence of contaminated (≥ 1 Colony Forming Units, CFU, blue) mosquitoes after reversible colonisation (0 CFU, white). Data represents mean ± SEM of three independent replicates where 34, 123 and 321 mosquitoes were analysed 0-2 days after emergence. Source Data are provided as a Source Data file.

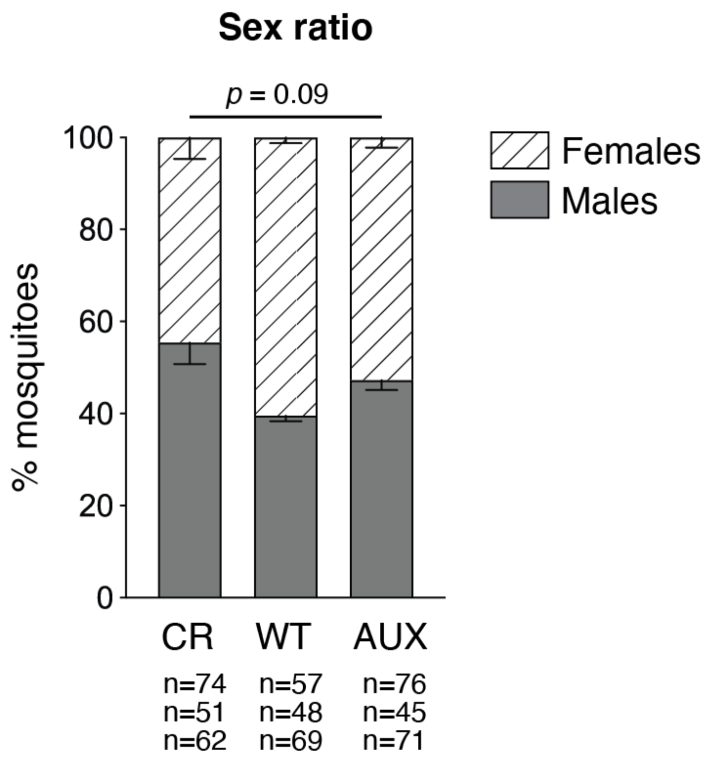

Figure S4. Sex ratio of conventionally reared and gnotobiotic mosquitoes. Ratio between adult females (striped bars) and males (full bars) emerged from conventionally-reared (CR), wild-type *E. coli* gnotobiotic (WT), and auxotrophic *E. coli* gnotobiotic larvae (AUX). Data show mean ± SEM of three independent replicates. The exact number of individuals analysed per condition and replicate is indicated in the figure. Statistical significance was determined with a generalized linear mixed model and least square means with Bonferroni correction. The exact *p* value is indicated in the figure. See Table S1 for detailed statistical information. Source Data are provided as a Source Data file. Data shown in Figure 1b and Figure S4 derive from the same experiments.

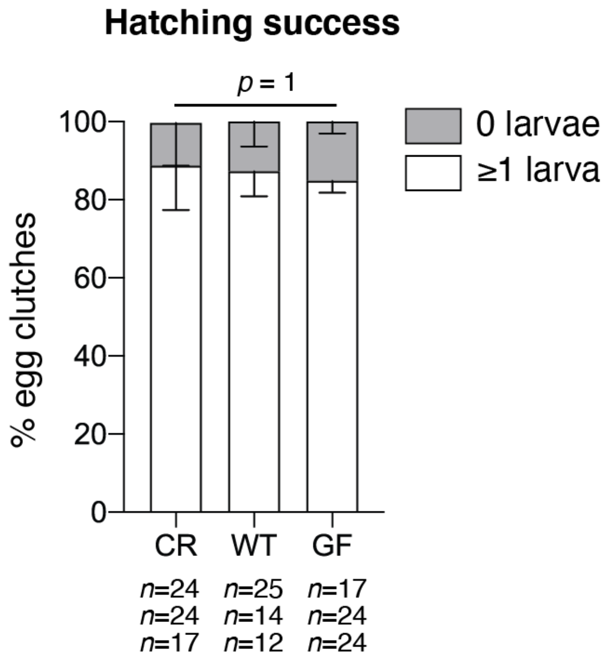

Figure S5. Percentage of egg clutches producing viable larvae (≥ 1 larva, white bars) and not originating larvae (0 larvae, grey bars) for mosquitoes conventionally reared (CR), reared on wild-type *E. coli* (WT) and reversibly colonised (GF). mean ± SEM). Data show mean ± SEM of three independent replicates. The exact number of individuals analysed per condition and replicate is indicated in the figure. Statistical significance was determined with a generalized linear mixed model and least square means with Bonferroni correction. The exact *p* value is indicated in the figure. See Table S1 for detailed statistical information. Source Data are provided as a Source Data file. Data shown in Figure 2d-e and Figure S5 derive from the same experiments.

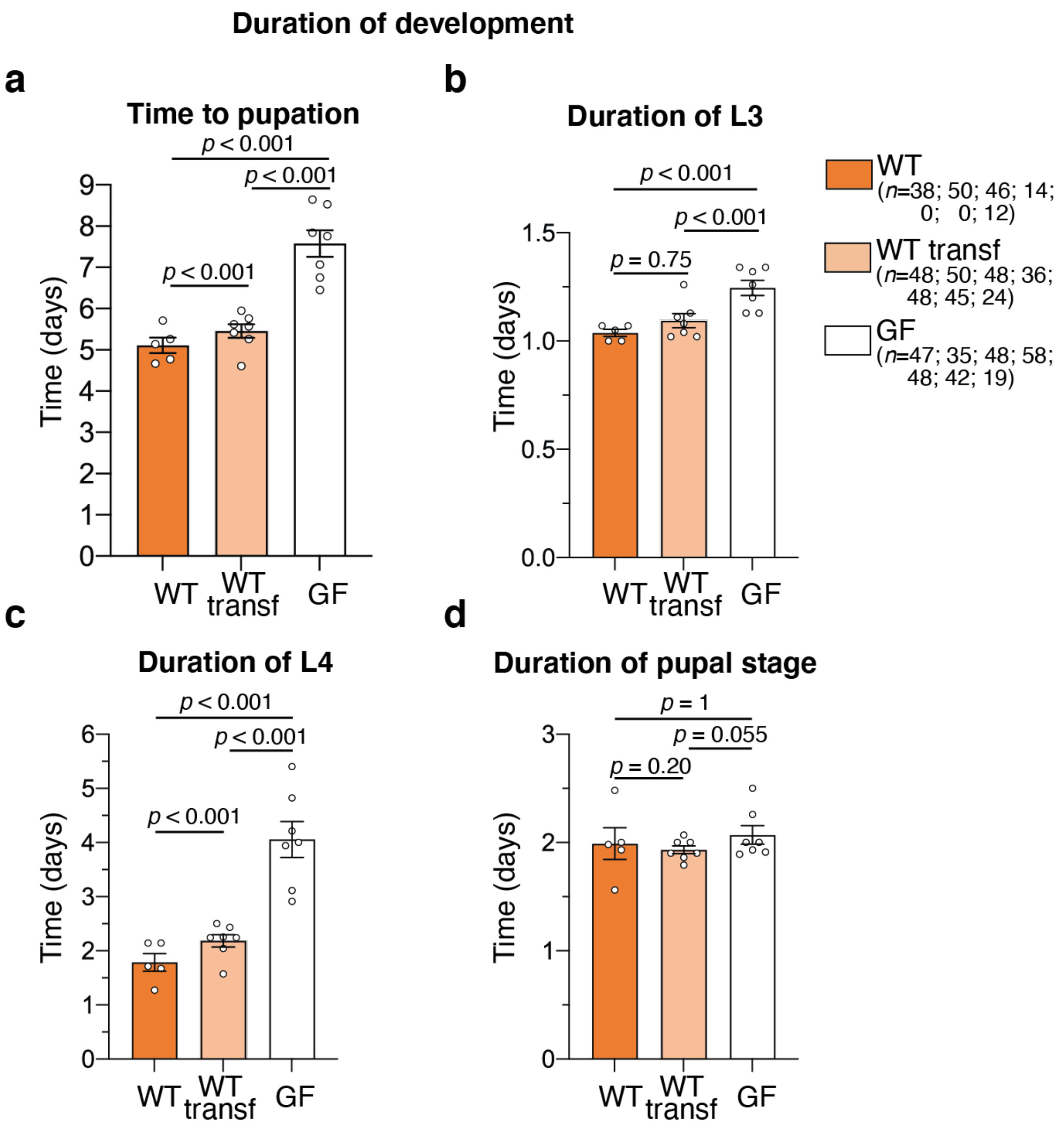

Figure S6. Duration of development of third instar larvae transferred in new sterile medium. Total time to pupation (**a**), duration of the third instar (L3, **b**), fourth instar (L4, **c**) and pupal stage (**d**) when continuously colonised (WT, orange), reared on wild-type *E. coli* and transferred in new rearing medium (WT transferred, light orange) and after becoming germ-free (GF, white). Data show mean ± SEM of five (WT) or seven (WT transferred and GF) independent replicates. The exact number of individuals analysed per condition and replicate is indicated in the figure. Statistical significance was determined with generalized linear mixed models and least square means with Bonferroni correction. Exact *p* values are indicated in the figure. See Table S1 for detailed statistical information. Source Data are provided as a Source Data file. Data shown in Figure 3c and Figure S6 derive from the same experiments.

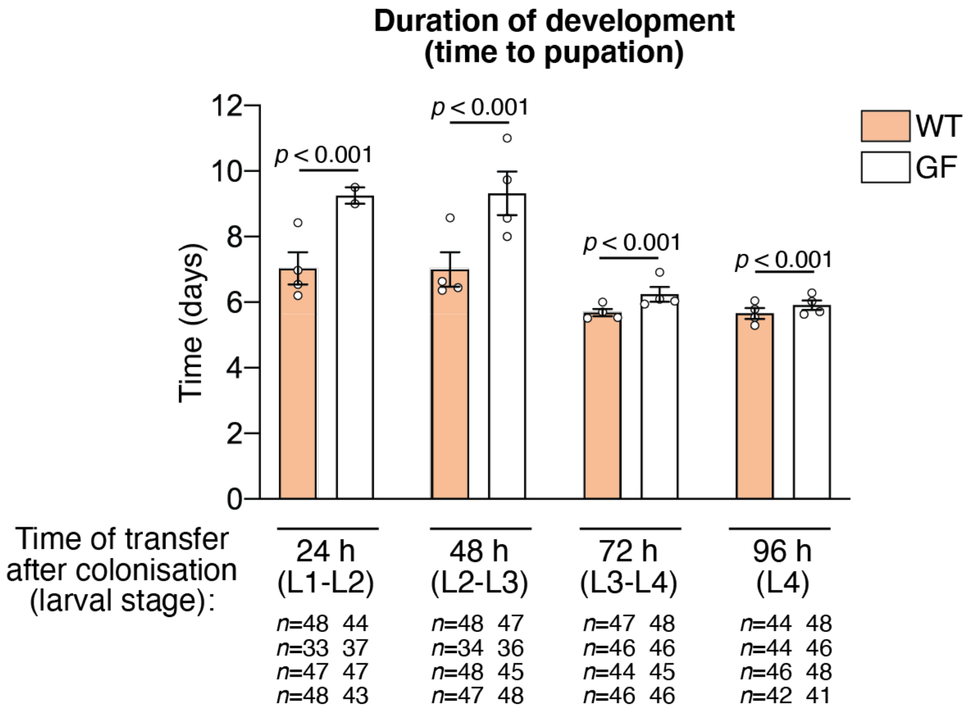

Figure S7. Duration of development of larvae transferred in new sterile medium at different time-points when reared on wild-type *E. coli* and transferred in new rearing medium (WT, light orange) and after becoming germ-free (GF, white). Data show mean ± SEM of four independent replicates. The exact number of individuals analysed per condition and replicate is indicated in the figure. Statistical significance was determined with generalized linear mixed models and least square means with Bonferroni correction. Exact *p* values are indicated in the figure. See Table S1 for detailed statistical information. Source Data are provided as a Source Data file. Data shown in Figure 3e and Figure S7 derive from the same experiments.

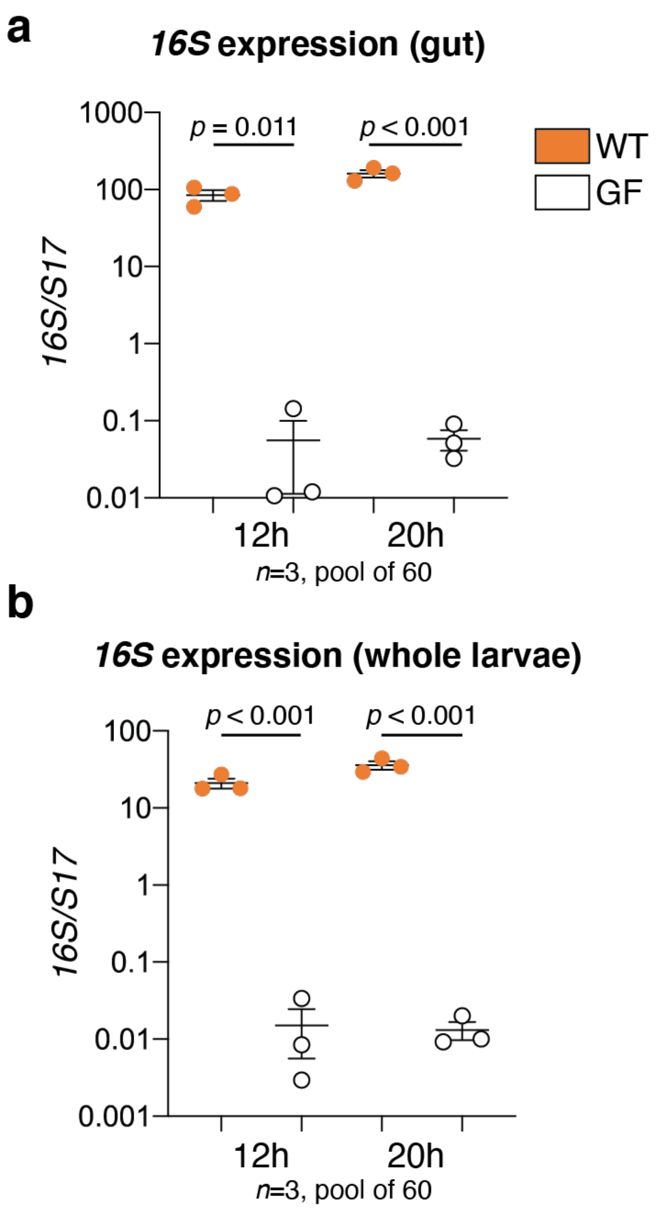

Figure S8. Quantification via qPCR of the bacterial load in guts (**a**) and whole larvae (**b**) of colonised and germ-free third-instar larvae at the 12 h and 20 h time-points. Data are expressed as the ratio between bacterial *16S* rRNA and *Ae. aegypti S17* genes in wild-type *E. coli*-carrying larvae (WT, orange) and in germ-free larvae (GF, white) and represents mean ± SEM of three independent replicates. The RNA samples were those analysed in the transcriptomic study (n = 60 larvae per condition per replicate). Statistical significance was determined with a generalized linear mixed model and least square means with Bonferroni correction. Exact *p* values are indicated in the figure. See Table S1 for detailed statistical information. Source Data are provided as a Source Data file.

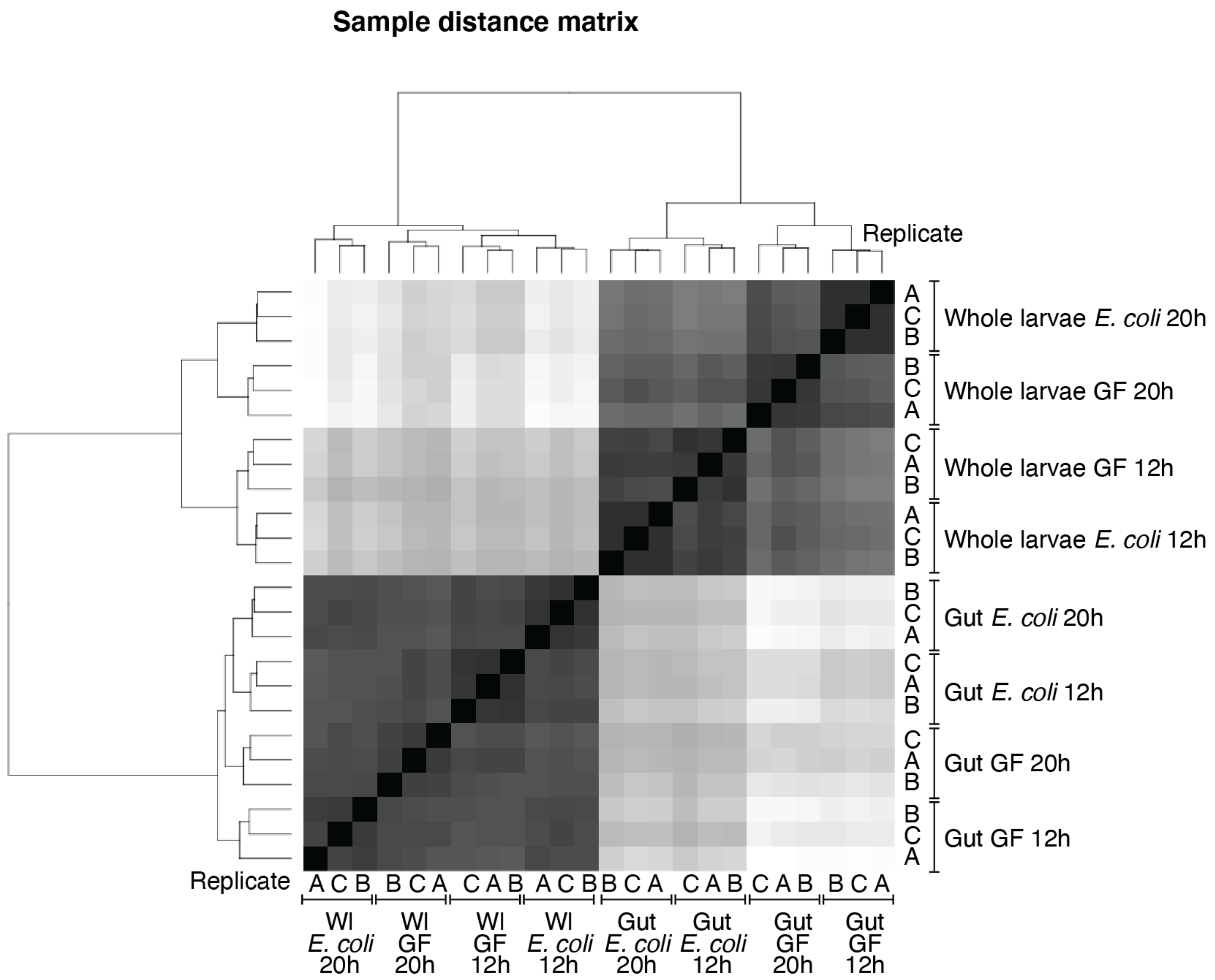

Figure S9. Sample distance matrix of gut and whole larvae (Wl) transcriptomic data at the 12 h and 20 h time-points in larvae reared on wild-type *E. coli* (WT) and after becoming germ-free (GF). Heatmap showing the regularized log transformation of the differential expression analysis data performed with DESeq2. Source Data are provided as a Source Data file.

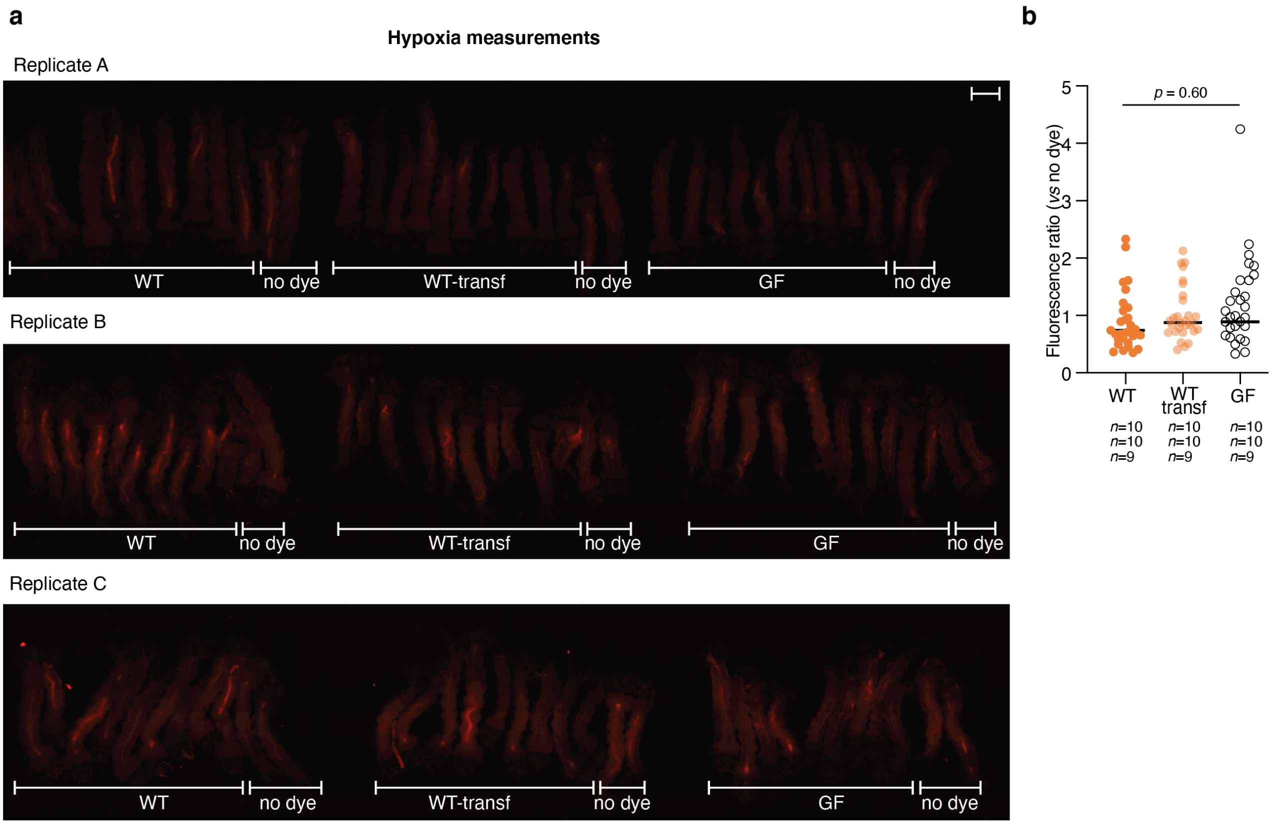

Figure S10. Hypoxia measurements on colonised and germ-free third-instar larvae 12 h after transfer. (**a**) Images show third-instar larvae gnotobiotic for wild-type *E. coli* before (WT) and after (WT transf) the transferring to new rearing medium and germ-free (GF) stained with the Image-iT Red Hypoxia Reagent at the 12 h time-point. For each condition, 9-10 larvae were stained with the dye, while 2 larvae were kept without dye as negative control for autofluorescence. For each larva, the ratio between the fluorescence intensity was normalised to the larval area. Scale bar: 1 mm. (**b**) Measurements of fluorescence intensity ratios with respect to no dye controls. Data show mean ± SEM of three independent replicates. The exact number of individuals analysed per condition and replicate is indicated in the figure. Statistical significance was determined with a generalized linear mixed model and least square means with Bonferroni correction. The exact *p* value is indicated in the figure. See Table S1 for detailed statistical information. Source Data are provided as a Source Data file.

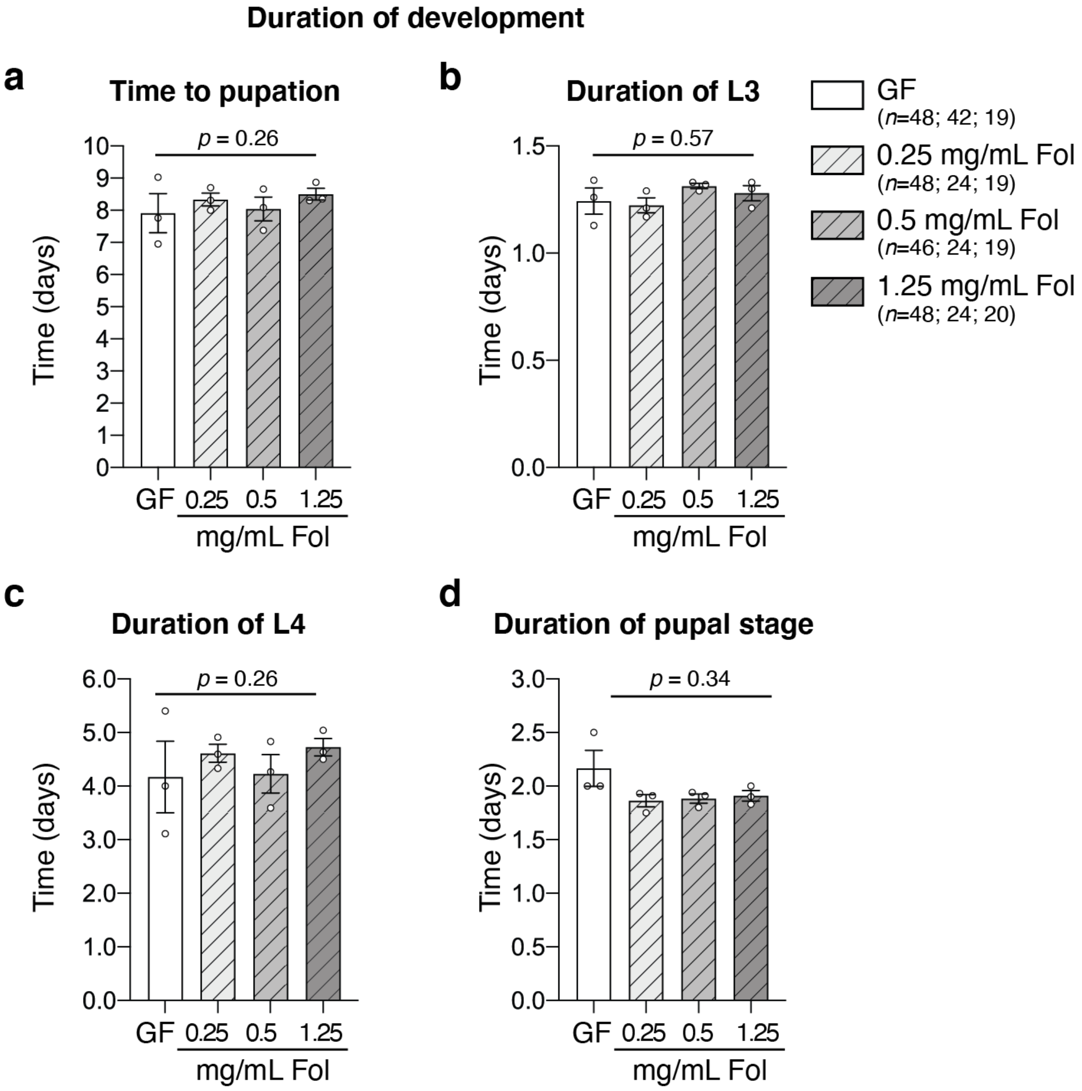

Figure S11. Total time to pupation (**a**), duration of the third instar (L3, **b**), fourth instar (L4, **c**) and pupal stage (**d**, mean ± SEM) of third-instar larvae in germ-free conditions (GF, white) or in germ-free conditions with folic acid (Fol) supplementation at different concentrations (light grey: 0.25 mg/mL folic acid; mid grey: 0.5 mg/mL folic acid; dark grey: 1.25 mg/mL folic acid). Data show mean ± SEM of three independent replicates. The exact number of individuals analysed per condition and replicate is indicated in the figure. Statistical significance was determined with generalized linear mixed models and least square means with Bonferroni correction. Exact *p* values are indicated in the figure. See Table S1 for detailed statistical information. Source Data are provided as a Source Data file. Data shown in Figure 6b and Figure S11 derive from the same experiments.

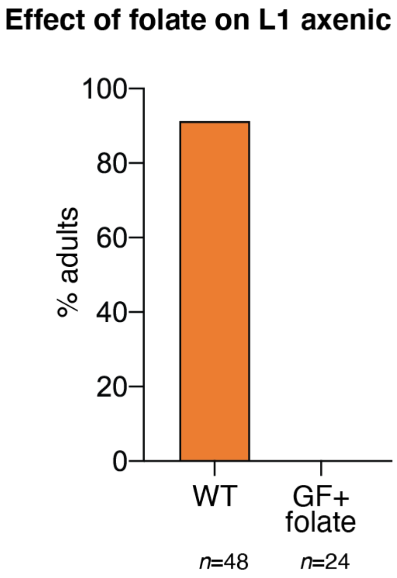

Figure S12. Effect of 1.25 mg/mL folate supplementation on the development of first instar axenic larvae (GF, n=24), compared to larvae colonised with WT *E. coli* (WT, n=48).

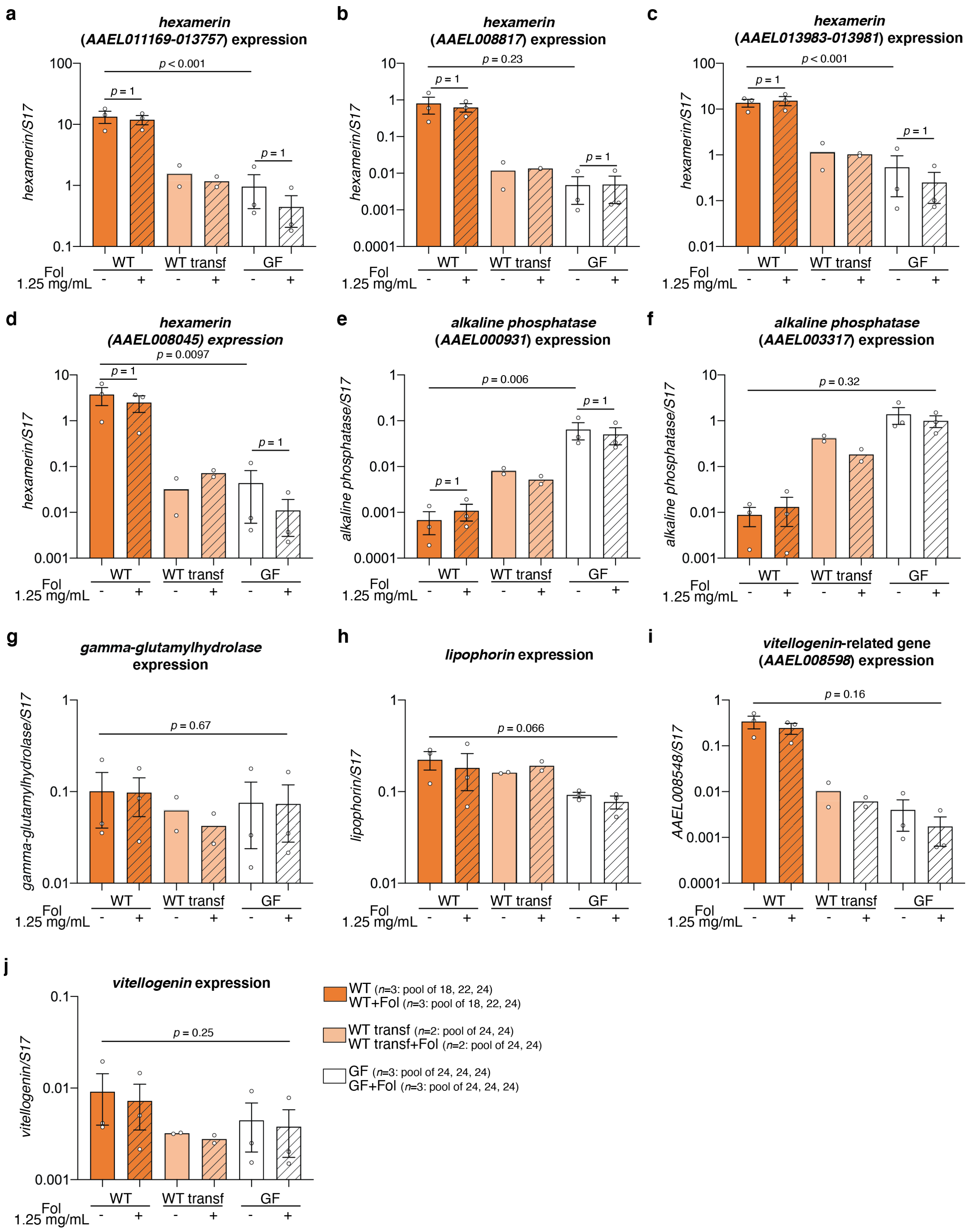

Figure S13. Effect of folic acid supplementation (1.25 mg/mL, striped bars) for 20 h on the expression of *hexamerins AAEL011169-AAEL013757* (**a**), *AAEL008817* (**b**), *AAEL013983-AAEL013981* (**c**), *AAEL008045* (**d**), *alkaline phosphatase AAEL000931* (**e**), *AAEL003317* (**f**), *gamma-glutamyl hydrolase* (**g**), *lipophorin* (**h**), *vitellogenin*-related gene *AAEL008598* (**i**), and *vitellogenin* (**j**) in larvae reared continuously with wild-type *E. coli* (WT, orange), transferred in new rearing medium after being reared with wild-type *E. coli* (WT transf, light orange) and after becoming germ-free (GF, white). Data show the mean

± SEM of three independent replicates except for expression data on WT transferred larvae which derive from two replicates. The exact number of individuals pooled per condition and replicate is indicated in the figure. Statistical significance was determined with generalized linear mixed models and least square means with Bonferroni correction. Exact *p* values are indicated in the figure. See Table S1 for detailed statistical information. Source Data are provided as a Source Data file.

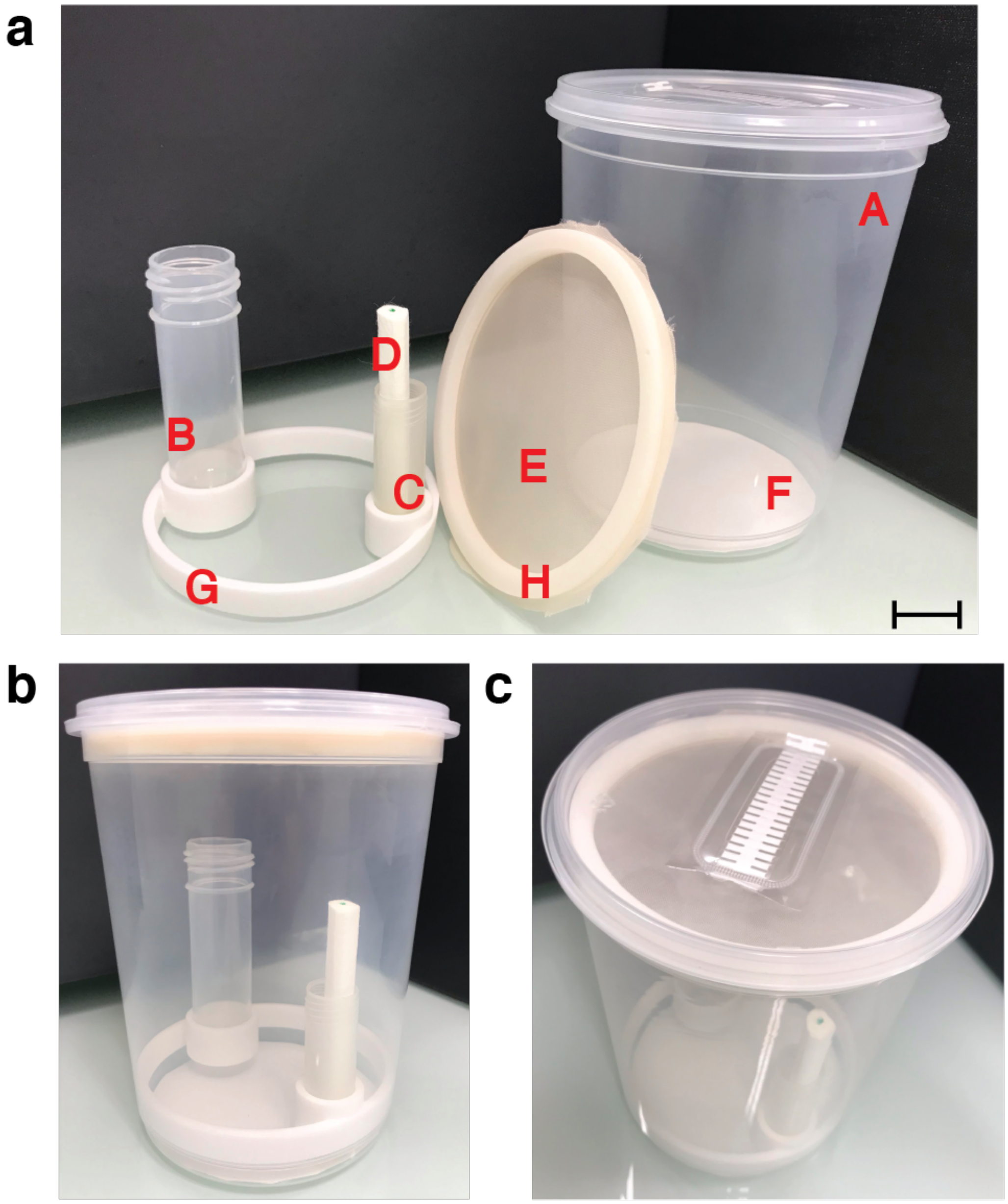

Figure S14. Preparation of sterile boxes for germ-free adult mosquitoes. (**a**) Material needed to set-up boxes: autoclavable polypropylene box for plant culture (**A**), larger autoclavable tube (**B**), smaller autoclavable tube (**C**), cotton roll (**D**), mosquito net (**E**), filter paper cut to fit the bottom of the box (**F**). The filter paper is fixed to the bottom of the box with adhesive tape to collect mosquito excreta. The larger tube will allocate pupae, while in the smaller one a cotton roll is placed for sugar feeding. Boxes are equipped with two components printed in autoclavable material (PA 2200, Shapeways): the first one is designed to hold the two tubes (**G**), while the other component consists of two clippable rings holding a disposable mosquito net in the middle (**H**). This second component was designed to avoid mosquito contamination through the upper filter and to allow mosquito blood-feeding in sterile conditions. (**b**-**c**) Two different views of the complete box set-up for axenic mosquito rearing. After autoclaving, a sterile 10% sucrose solution is added to the smaller tube with the cotton roll. Scale bar in (**a**) corresponds to 2 cm.

Table S1. Statistical information

Analysis	Response variable	Predictor	Random effect	Sample size	Test result	Comparisons
**Figure 1b. Developmental success**						
Glmm (binomial)	% adults	Rearing condition	Replicate	Replicate A: AUX (n=76); CR (n=74); WT(n=57)<br>Replicate B: AUX (n=45); CR (n=51); WT (n=48)<br>Replicate C: AUX (n=71); CR (n=62); WT (n=69)	$F_{2,16} = 7.9$, $p < 0.001$	lsmeans (Bonferroni correction):<br>AUX vs CR: $p < 0.001$<br>AUX vs WT: $p = 0.87$<br>CR vs WT: $p = 0.02$
**Figure S3. Sex ratio**						
Glmm (binomial)	% females	Rearing condition	Replicate	Replicate A: AUX (n=76); CR (n=74); WT(n=57)<br>Replicate B: AUX (n=45); CR (n=51); WT (n=48)<br>Replicate C: AUX (n=71); CR (n=62); WT (n=69)	$F_{2,8.3} = 4.2$, $p = 0.09$	/
**Figure 1c. Colonisation dynamics in larvae/pupae**						
Glmm	CFU (L3) day 3	Bacterium	Replicate	Replicate A: AUX (n=12); WT(n=12)<br>Replicate B: AUX (n=12); WT(n=12)<br>Replicate C: AUX (n=18); WT(n=12)	$F_{1,9e6} = 0.14$, $p = 0.71$	/
Glmm	CFU(L4) day 4	Bacterium	Replicate	Replicate A: AUX (n=12); WT(n=12)<br>Replicate B: AUX (n=12); WT(n=11)<br>Replicate C: AUX (n=18); WT(n=12)	$F_{1,1e8} = 4.5$, $p = 0.033$	/
Glmm	CFU(L4) day 5	Bacterium	Replicate	Replicate A: AUX (n=12); WT(n=12)<br>Replicate B: AUX (n=12); WT(n=12)<br>Replicate C: AUX (n=18); WT(n=12)	$F_{1,1e8} = 44$, $p < 0.001$	/
Glmm	CFU(pupae) day 6	Bacterium	Replicate	Replicate A: AUX (n=12); WT(n=12)<br>Replicate B: AUX (n=12); WT(n=8)<br>Replicate C: AUX (n=13); WT(n=12)	$F_{1,65} = 66$, $p < 0.001$	/
**Figure 1d. Reversible colonisation**						
Glmm	CFU (adults) day 1	Bacterium	Replicate	Replicate A: AUX (n=12); WT(n=12)<br>Replicate B: AUX (n=12); WT(n=12)<br>Replicate C: AUX (n=18); WT(n=6)	$F_{1,64} = 5.9$, $p = 0.018$	/
Glmm	CFU (adults) day 2	Bacterium	Replicate	Replicate A: AUX (n=12); WT(n=12)<br>Replicate B: AUX (n=12); WT(n=12)<br>Replicate C: AUX (n=18); WT(n=6)	$F_{1,64} = 5.1$, $p = 0.027$	/
Glmm	CFU (adults) day 3	Bacterium	Replicate	Replicate A: AUX (n=12); WT(n=12)<br>Replicate B: AUX (n=12); WT(n=12)<br>Replicate C: AUX (n=18); WT(n=6)	$F_{1,64} = 4.0$, $p = 0.049$	/
**Figure 1e. Bacterial DNA detection via qPCR**						
	Pools of sugar-fed midguts			Replicate A: AUX (n=10); CR (n=10); WT(n=10)<br>Replicate B: AUX (n=10); CR (n=10); WT(n=10)<br>Replicate C: AUX (n=10); CR (n=10); WT(n=10)		
	Pools of blood-fed midguts			Replicate A: AUX (n=4); CR (n=5); WT(n=10)<br>Replicate B: AUX (n=6); CR (n=5); WT(n=4)<br>Replicate C: AUX (n=5); CR (n=6); WT(n=4)		
	Pools of sugar-fed whole mosquitoes			Replicate A: AUX (n=15); CR (n=12); WT(n=14)<br>Replicate B: AUX (n=12); CR (n=16); WT(n=15)<br>Replicate C: AUX (n=16); CR (n=10); WT(n=16)		

Analysis	Response variable	Predictor	Random effect	Sample size	Test result	Comparisons
**Figure S4a. Bacterial concentration without larvae**						
Glmm	CFU (WT)	Time	Replicate	Replicate A: WT (n=1)<br>Replicate B: WT (n=1)<br>Replicate C: WT (n=1)<br>Replicate D: WT (n=2)	$F_{6,Inf} = 3.4$, $p = 0.002$	lsmeans (Bonferroni correction):<br>day 0 vs day 1: $p = 0.24$<br>day 0 vs day 2: $p = 0.016$<br>day 0 vs day 3: $p = 0.0039$<br>day 0 vs day 4: $p = 0.019$<br>day 0 vs day 5: $p = 0.044$<br>day 0 vs day 6: $p = 1$<br>day 1 vs day 2: $p = 1$<br>day 2 vs day 3: $p = 1$<br>day 3 vs day 4: $p = 1$<br>day 4 vs day 5: $p = 1$<br>day 5 vs day 6: $p = 1$
Glmm	CFU (AUX)	Time	Replicate	Replicate A: AUX (n=1)<br>Replicate B: AUX (n=1)<br>Replicate C: AUX (n=1)<br>Replicate D: AUX (n=1)	$F_{6,Inf} = 3.0$, $p = 0.006$	lsmeans (Bonferroni correction):<br>day 0 vs day 1: $p = 0.74$<br>day 0 vs day 2: $p = 0.028$<br>day 0 vs day 3: $p = 0.015$<br>day 0 vs day 4: $p = 0.0098$<br>day 0 vs day 5: $p = 0.051$<br>day 0 vs day 6: $p = 0.30$<br>day 1 vs day 2: $p = 1$<br>day 2 vs day 3: $p = 1$<br>day 3 vs day 4: $p = 1$<br>day 4 vs day 5: $p = 1$<br>day 5 vs day 6: $p = 1$
Glmm	CFU (day 0)	Bacterium	Replicate		$F_{1,1e8} = 0.044$, $p = 0.83$	/
Glmm	CFU (day 1)	Bacterium	Replicate		$F_{1,6e6} = 0.081$, $p = 0.78$	/
Glmm	CFU (day 2)	Bacterium	Replicate		$F_{1,5802} = 0.84$, $p = 0.36$	/
Glmm	CFU (day 3)	Bacterium	Replicate		$F_{1,9e4} = 1.8$, $p = 0.18$	/
Glmm	CFU (day 4)	Bacterium	Replicate		$F_{1,6e47} = 1.2$, $p = 0.27$	/
Glmm	CFU (day 5)	Bacterium	Replicate		$F_{1,1e5} = 5.7$, $p = 0.017$	/
Glmm	CFU (day 6)	Bacterium	Replicate		$F_{1,2e4} = 0.85$, $p = 0.35$	/
**Figure S4b. Bacterial concentration with larvae**						
Glmm	CFU (WT)	Time	Replicate	Replicate A: WT (n=1)<br>Replicate B: WT (n=1)<br>Replicate C: WT (n=1)<br>Replicate D: WT (n=4)	$F_{6,Inf} = 14.16$, $p < 0.001$	lsmeans (Bonferroni correction):<br>day 0 vs day 1: $p = 0.0042$<br>day 0 vs day 2: $p < 0.001$<br>day 0 vs day 3: $p < 0.001$<br>day 0 vs day 4: $p < 0.001$<br>day 0 vs day 5: $p < 0.001$<br>day 0 vs day 6: $p < 0.001$<br>day 1 vs day 2: $p = 1$<br>day 2 vs day 3: $p = 1$<br>day 3 vs day 4: $p = 0.015$<br>day 4 vs day 5: $p = 1$

Analysis	Response variable	Predictor	Random effect	Sample size	Test result	Comparisons
						day 5 vs day 6: $p = 1$
Glmm	CFU (AUX)	Time	Replicate	Replicate A: AUX (n=1)<br>Replicate B: AUX (n=1)<br>Replicate C: AUX (n=1)<br>Replicate D: AUX (n=5)	$F_{6,Inf} = 16.4, p < 0.001$	lsmeans (Bonferroni correction):<br>day 0 vs day 1: $p = 0.0064$<br>day 0 vs day 2: $p = 0.098$<br>day 0 vs day 3: $p = 0.0090$<br>day 0 vs day 4: $p < 0.001$<br>day 0 vs day 5: $p < 0.001$<br>day 0 vs day 6: $p < 0.001$<br>day 1 vs day 2: $p = 1$<br>day 2 vs day 3: $p = 1$<br>day 3 vs day 4: $p = 0.0074$<br>day 4 vs day 5: $p = 1$<br>day 5 vs day 6: $p = 1$
Glmm	CFU (day 0)	Bacterium	Replicate		$F_{1,7e10} = 4.6, p = 0.83$	/
Glmm	CFU (day 1)	Bacterium	Replicate		$F_{1,2e6} = 0.062, p = 0.80$	/
Glmm	CFU (day 2)	Bacterium	Replicate		$F_{1,8e5} = 2.4, p = 0.12$	/
Glmm	CFU (day 3)	Bacterium	Replicate		$F_{1,1e7} = 0.67, p = 0.41$	/
Glmm	CFU (day 4)	Bacterium	Replicate		$F_{1,2e6} = 0.09, p = 0.76$	/
Glmm	CFU (day 5)	Bacterium	Replicate		$F_{1,4e9} = 15, p < 0.001$	/
Glmm	CFU (day 6)	Bacterium	Replicate		$F_{1,5e6} = 39, p < 0.001$	/
**Figure 2a. Duration of development**						
Glmm	Duration (L1 to L2)	Rearing condition	Replicate	Replicate A: AUX (n=76); CR (n=74); WT(n=57)<br>Replicate B: AUX (n=45); CR (n=51); WT (n=48)<br>Replicate C: AUX (n=71); CR (n=62); WT (n=69)	$F_{2,550} = 18, p < 0.001$	lsmeans (Bonferroni correction):<br>AUX vs CR: $p < 0.001$<br>AUX vs WT: $p = 0.0010$<br>CR vs WT: $p = 0.095$
Glmm	Duration (L1 to L3)	Rearing condition	Replicate		$F_{2,526} = 3.1, p = 0.044$	lsmeans (Bonferroni correction):<br>AUX vs CR: $p = 0.13$<br>AUX vs WT: $p = 0.07$<br>CR vs WT: $p = 1$
Glmm	Duration (L1 to L4)	Rearing condition	Replicate		$F_{2,503} = 6.9, p = 0.0010$	lsmeans (Bonferroni correction):<br>AUX vs CR: $p = 0.72$<br>AUX vs WT: $p < 0.001$<br>CR vs WT: $p = 0.051$
Glmm	Duration (L1 to pupa)	Rearing condition	Replicate		$F_{2,475} = 2.9, p = 0.057$	/
Glmm	Duration (L1 to adult)	Rearing condition	Replicate		$F_{2,467} = 1.0, p = 0.37$	/
**Figure 2b. Larval length**						
Glmm	Length	Rearing condition	Replicate	Replicate A: AUX (n=39); CR (n=28); WT(n=55)<br>Replicate B: AUX (n=56); CR (n=32); WT (n=42)<br>Replicate C: AUX (n=30); CR (n=36); WT (n=36)<br>Replicate D: AUX (n=61); CR (n=27); WT (n=45)	$F_{2,481} = 2.5, p = 0.083$	/
**Figure 2c. Wing length**						

Analysis	Response variable	Predictor	Random effect	Sample size	Test result	Comparisons
Glmm	Length (females)	Rearing condition	Replicate	Replicate A: AUX (n=11); CR (n=16); WT(n=20)<br>Replicate B: AUX (n=12); CR (n=12); WT (n=7)<br>Replicate C: AUX (n=20); CR (n=8); WT (n=20)<br>Replicate D: AUX (n=20); CR (n=16); WT (n=19)	$F_{2,176}$ = 1.7, p = 0.19	/
Glmm	Length (males)	Rearing condition	Replicate	Replicate A: AUX (n=17); CR (n=16); WT(n=20)<br>Replicate B: AUX (n=20); CR (n=15); WT (n=20)<br>Replicate C: AUX (n=8); CR (n=18); WT (n=18)<br>Replicate D: AUX (n=13); CR (n=20); WT (n=20)	$F_{2,199}$ = 1.9, p = 0.14	/
**Figure 2d. Egg laying**						
Glmm (binomial)	Egg laying mosquitoes	Rearing condition	Replicate	Replicate A: GF (n=17); CR (n=24); WT(n=25)<br>Replicate B: GF (n=24); CR (n=24); WT (n=14)<br>Replicate C: GF (n=24); CR (n=17); WT (n=12)	$F_{2,27}$ = 14, p < 0.001	lsmeans (Bonferroni correction):<br>GF vs CR: p < 0.001<br>GF vs WT: p = 1<br>CR vs WT: p < 0.001
Glmm (binomial)	Egg laying mosquitoes (WT)	Contamination	Replicate	Replicate A: WT cont (n=7); WT non-cont (n=18)<br>Replicate B: WT cont (n=5); WT non-cont (n=9)<br>Replicate C: WT cont (n=6); WT non-cont (n=6)	$F_{2,2}$ = 1.7, p = 0.22	/
**Figure 2e. Clutch size**						
Glmm	Number of eggs	Rearing condition	Replicate	See egg laying (Figure 2d)	$F_{2,167}$ = 9.4, p < 0.001	lsmeans (Bonferroni correction):<br>GF vs CR: p = 0.0089<br>GF vs WT: p = 0.78<br>CR vs WT: p < 0.001
Glmm	Number of eggs (WT)	Contamination	Replicate	See egg laying (Figure 2d)	$F_{2,48}$ = 0.11, p = 0.74	/
**Figure S5. Hatching success**						
Glmm (binomial)	Positive hatching	Rearing condition	Replicate	See egg laying (Figure 2d)	$F_{2,0.1}$ = 0.062, p = 1	/
Glmm (binomial)	Positive hatching (WT)	Contamination	Replicate	See egg laying (Figure 2d)	$F_{1,0.6}$ = 0.62, p = 0.43	/
**Figure 2f. Lifespan**						
Cox model	Survival (females)	Bacterium	/	Replicate A: GF (n=22); WT(n=25)<br>Replicate B: GF (n=21); WT (n=25)<br>Replicate C: GF (n=24); WT (n=19)	z = 1.2, p = 0.24<br>0.95 CI (0.87-1.7)	/
Cox model	Survival (males)	Bacterium	/	Replicate A: GF (n=28); WT(n=28)<br>Replicate B: GF (n=19); WT (n=22)<br>Replicate C: GF (n=22); WT (n=22)	z = -0.31, p = 0.76<br>0.95 CI (0.68-1.3)	/
**Figure 3b. Bacterial load after transfer (third instar larvae)**						
Glmm	CFU (NT)	Bacterium	Replicate	Replicate A: AUX (n=6); WT(n=12)<br>Replicate B: AUX (n=6); WT (n=6)<br>Replicate C: AUX (n=6); WT (n=6)	$F_{1,3e5}$ = 6.2, p = 0.013	/
Glmm	CFU (2 h)	Bacterium	Replicate	Replicate A: AUX (n=6); WT(n=12)<br>Replicate B: AUX (n=6); WT (n=6)<br>Replicate C: AUX (n=6); WT (n=6)	$F_{1,39}$ = 0.60, p = 0.44	/
Glmm	CFU (5 h)	Bacterium	Replicate	Replicate A: AUX (n=6); WT(n=11)<br>Replicate B: AUX (n=6); WT (n=6)	$F_{1,37}$ = 42, p < 0.001	/

Analysis	Response variable	Predictor	Random effect	Sample size	Test result	Comparisons
				Replicate C: AUX (n=6); WT (n=6)		
Glmm	CFU (12 h)	Bacterium	Replicate	Replicate A: AUX (n=6); WT(n=6)<br>Replicate B: AUX (n=6); WT (n=8)<br>Replicate C: AUX (n=6); WT (n=6)	$F_{1,34} = 19, p < 0.001$	/
Glmm	CFU (20 h)	Bacterium	Replicate	Replicate A: AUX (n=6); WT(n=6)<br>Replicate B: AUX (n=6); WT (n=8)<br>Replicate C: AUX (n=6); WT (n=6)	$F_{1,8e4} = 1.2, p = 0.26$	/
Glmm	CFU (WT)	Time-point	Replicate		$F_{4,3e4} = 1.8, p = 0.12$	/
Glmm	CFU (AUX)	Time-point	Replicate		$F_{4,83} = 29, p < 0.001$	lsmeans (Bonferroni correction):<br>NT vs 2 h: $p < 0.001$<br>NT vs 5 h: $p < 0.001$<br>NT vs 12 h: $p < 0.001$<br>NT vs 20 h: $p < 0.001$
**Figure 3c. Development success of third-instar larvae**						
Glmm (binomial)	% L4	Rearing condition	Replicate	Replicate A: AUX (n=47); WT(n=38); WT transf (n=48)<br>Replicate B: AUX (n=35); WT(n=50); WT transf (n=50)<br>Replicate C: AUX (n=48); WT(n=46); WT transf (n=48)<br>Replicate D: AUX (n=58); WT(n=14); WT transf (n=36)<br>Replicate E: AUX (n=48); WT(n=0); WT transf (n=48)<br>Replicate F: AUX (n=42); WT(n=0); WT transf (n=45)<br>Replicate G: AUX (n=19); WT(n=12); WT transf (n=24)	$F_{2,0.001} = 8*10^{-4}, p = 1$	/
Glmm (binomial)	% pupae	Rearing condition	Replicate		$F_{2,84} = 42, p < 0.001$	lsmeans (Bonferroni correction):<br>GF vs WT: $p < 0.001$<br>GF vs WT transf: $p = 0.004$<br>WT vs WT transf: $p < 0.001$
Glmm (binomial)	% adults	Rearing condition	Replicate		$F_{2,111} = 55, p < 0.001$	lsmeans (Bonferroni correction):<br>GF vs WT: $p < 0.001$<br>GF vs WT transf: $p < 0.001$<br>WT vs WT transf: $p < 0.001$
**Figure S6. Duration of development**						
Glmm	Time to pupa	Rearing condition	Replicate	See development success of third-instar larvae (Figure 3c)	$F_{2,370} = 175, p < 0.001$	lsmeans (Bonferroni correction):<br>GF vs WT: $p < 0.001$<br>GF vs WT transf: $p < 0.001$<br>WT vs WT transf: $p < 0.001$
Glmm	Time (L3)	Rearing condition	Replicate		$F_{2,696} = 16, p < 0.001$	lsmeans (Bonferroni correction):<br>GF vs WT: $p < 0.001$<br>GF vs WT transf: $p < 0.001$<br>WT vs WT transf: $p = 0.75$
Glmm	Time (L4)	Rearing condition	Replicate		$F_{2,373} = 181, p < 0.001$	lsmeans (Bonferroni correction):<br>GF vs WT: $p < 0.001$<br>GF vs WT transf: $p < 0.001$<br>WT vs WT transf: $p < 0.001$
Glmm	Time (pupal stage)	Rearing condition	Replicate		$F_{2,345} = 3.3, p = 0.039$	lsmeans (Bonferroni correction):<br>GF vs WT: $p = 1$<br>GF vs WT transf: $p = 0.055$<br>WT vs WT transf: $p = 0.20$
**Figure 3d. Bacterial load 16 h after transfer**						
Glmm	CFU (transf 24 h)	Bacterium	Replicate	Replicate A: AUX (n=6); WT (n=6)	$F_{1,32} = 20, p < 0.001$	/

Analysis	Response variable	Predictor	Random effect	Sample size	Test result	Comparisons
				Replicate B: AUX (n=6); WT (n=6)<br>Replicate C: AUX (n=6); WT (n=6)		
Glmm	CFU (transf 48 h)	Bacterium	Replicate	Replicate A: AUX (n=6); WT (n=6)<br>Replicate B: AUX (n=6); WT (n=6)<br>Replicate C: AUX (n=6); WT (n=6)	$F_{1,32} = 22$, $p < 0.001$	/
Glmm	CFU (transf 72 h)	Bacterium	Replicate	Replicate A: AUX (n=6); WT (n=6)<br>Replicate B: AUX (n=6); WT (n=6)<br>Replicate C: AUX (n=6); WT (n=6)	$F_{1,34} = 11$, $p = 0.0021$	/
Glmm	CFU (transf 96 h)	Bacterium	Replicate	Replicate A: AUX (n=6); WT (n=6)<br>Replicate B: AUX (n=6); WT (n=6)<br>Replicate C: AUX (n=6); WT (n=6)	$F_{1,4e35} = 4.4$, $p = 0.036$	/
**Figure 3e. Development success after transfer at different time-points**						
Glmm (binomial)	% adults (transf 24 h)	Bacterium	Replicate	Replicate A: AUX (n=44); WT (n=48)<br>Replicate B: AUX (n=37); WT (n=33)<br>Replicate C: AUX (n=47); WT (n=47)<br>Replicate D: AUX (n=43); WT (n=48)	$F_{1,88} = 88$, $p < 0.001$	/
Glmm (binomial)	% adults (transf 48 h)	Bacterium	Replicate	Replicate A: AUX (n=47); WT (n=48)<br>Replicate B: AUX (n=36); WT (n=34)<br>Replicate C: AUX (n=45); WT (n=48)<br>Replicate D: AUX (n=48); WT (n=47)	$F_{1,67} = 67$, $p < 0.001$	/
Glmm (binomial)	% adults (transf 72 h)	Bacterium	Replicate	Replicate A: AUX (n=48); WT (n=47)<br>Replicate B: AUX (n=46); WT (n=46)<br>Replicate C: AUX (n=45); WT (n=44)<br>Replicate D: AUX (n=46); WT (n=46)	$F_{1,11} = 11$, $p < 0.001$	/
Glmm (binomial)	% adults (transf 96 h)	Bacterium	Replicate	Replicate A: AUX (n=48); WT (n=44)<br>Replicate B: AUX (n=46); WT (n=44)<br>Replicate C: AUX (n=48); WT (n=46)<br>Replicate D: AUX (n=41); WT (n=42)	$F_{1,0.5} = 0.49$, $p = 0.48$	/
Glmm (binomial)	% dead vs blocked (transf 24 h)	Bacterium	Replicate	See % adults (transf 24 h)	$F_{1,13} = 13$, $p < 0.001$	/
Glmm (binomial)	% dead vs blocked (transf 48 h)	Bacterium	Replicate	See % adults (transf 48 h)	$F_{1,1} = 1.2$, $p = 0.28$	/
Glmm (binomial)	% dead vs blocked (transf 72 h)	Bacterium	Replicate	See % adults (transf 72 h)	$F_{1,2} = 2.1$, $p =0.18$	/
Glmm (binomial)	% dead *vs* blocked (transf 96 h)	Bacterium	Replicate	See % adults (transf 96 h)	$F_{1,0.4} = 0.41$, $p = 0.53$	/
**Figure S7. Duration of development after transfer**						
Glmm	Time to pupation (transf 24 h)	Bacterium	Replicate	See % adults (transf 24 h, Figure 3e)	$F_{1,143} = 11$, $p < 0.001$	/
Glmm	Time to pupation (transf 48 h)	Bacterium	Replicate	See % adults (transf 48 h, Figure 3e)	$F_{1,116} = 67$, $p < 0.001$	/
Glmm	Time to pupation (transf 72 h)	Bacterium	Replicate	See % adults (transf 72 h, Figure 3e)	$F_{1,279} = 24$, $p < 0.001$	/

Analysis	Response variable	Predictor	Random effect	Sample size	Test result	Comparisons
Glmm	Time to pupation (transf 96 h)	Bacterium	Replicate	See % adults (transf 96 h, Figure 3e)	$F_{1,330} = 13, p < 0.001$	/
**Figure 4a. CFU after transfer (breeding site water)**						
Glmm	CFU NT vs 2-5 h (transf 48 h)	Transfer	Replicate	Replicate A: NT (n=6); TR-2h (n=5); TR-5h (n=6)<br>Replicate B: NT (n=6); TR-2h (n=6); TR-5h (n=6)	$F_{2,31} = 9.7, p < 0.001$	lsmeans (Bonferroni correction):<br>NT vs 2 h: $p = 0.0020$<br>NT vs 5 h: $p = 0.0018$<br>2 h vs 5 h: $p = 1$
Glmm	CFU NT-24 h vs 24 h (transf 48 h)	Transfer	Replicate	Replicate A: NT-24h (n=6); TR-24h (n=6)<br>Replicate B: NT-24h (n=6); TR-24h (n=6)	$F_{1,2e20} = 0.85, p = 0.36$	/
**Figure 4b. CFU after transfer (breeding site water)**						
Glmm	CFU NT vs 2-5 h (transf 72 h)	Transfer	Replicate	Replicate A: NT (n=6); TR-2h (n=6); TR-5h (n=6)<br>Replicate B: NT (n=6); TR-2h (n=6); TR-5h (n=6)<br>Replicate C: NT (n=6); TR-2h (n=6); TR-5h (n=6)	$F_{2,49} = 20, p < 0.001$	lsmeans (Bonferroni correction):<br>NT vs 2 h: $p < 0.001$<br>NT vs 5 h: $p < 0.001$<br>2 h vs 5 h: $p = 1$
Glmm	CFU NT-24 h vs 24 h (transf 72 h)	Transfer	Replicate	Replicate A: NT-24h (n=6); TR-24h (n=6)<br>Replicate B: NT-24h (n=6); TR-24h (n=6)<br>Replicate C: NT-24h (n=6); TR-24h (n=6)	$F_{1,3e5} = 1.7, p = 0.18$	/
**Figure 4c. Development success after transfer (breeding site water)**						
Glmm (binomial)	% adults (transf 48 h)	Transfer	Replicate	Replicate A: NT (n=44); TR-48 (n=44)<br>Replicate B: NT (n=40); TR-48 (n=39)<br>Replicate C: NT (n=50); TR-48 (n=44)	$F_{1,14} = 14, p < 0.001$	/
Glmm (binomial)	% dead vs blocked (transf 48 h)	Transfer	Replicate		$F_{1,17} = 17, p = 0.0081$	/
**Figure 4d. Development success after transfer (breeding site water)**						
Glmm (binomial)	% adults (transf 72 h)	Transfer	Replicate	Replicate A: NT (n=40); TR-72 (n=43)<br>Replicate B: NT (n=43); TR-72 (n=44)<br>Replicate C: NT (n=36); TR-72 (n=37)<br>Replicate D: NT (n=44); TR-72 (n=46)<br>Replicate E: NT (n=29); TR-72 (n=39)<br>Replicate F: NT (n=40); TR-72 (n=45)<br>Replicate G: NT (n=50); TR-72 (n=46)	$F_{1,9} = 9.3, p = 0.0023$	/
Glmm (binomial)	% dead vs blocked (transf 72 h)	Transfer	Replicate		$F_{1,22} = 22, p < 0.001$	/
**Figure S8. *16S* qPCR on sequenced samples**						
Glmm	Log_2 Ratio (gut 12 h)	Bacterium	Replicate	Replicate A: GF (n=1, pool of 60); WT (n=1, pool of 60)<br>Replicate B: GF (n=1, pool of 60); WT (n=1, pool of 60)<br>Replicate C: GF (n=1, pool of 60); WT (n=1, pool of 60)	$F_{1,2} = 92, p = 0.011$	/
Glmm	Log_2 Ratio (gut 20 h)	Bacterium	Replicate		$F_{1,4} = 631, p < 0.001$	/
Glmm	Log_2 Ratio (wl 12 h)	Bacterium	Replicate		$F_{1,4} = 114, p < 0.001$	/
Glmm	Log_2 Ratio (wl 20 h)	Bacterium	Replicate		$F_{1,2} = 2704, p < 0.001$	/
**Figure 6b. Development success with folate**						
Glmm (binomial)	% L4	Folate concentration	Replicate	Replicate A:<br>GF (n=48); 0.25 FOL (n=48); 0.5 FOL (n=46); 1.25 FOL (n=48)<br>Replicate B:<br>GF (n=42); 0.25 FOL (n=24); 0.5 FOL (n=24); 1.25 FOL (n=24)<br>Replicate C:	$F_{3,7e\text{-}11} = 0, p = 1$	/
Glmm (binomial)	% pupae	Folate concentration	Replicate		$F_{3,51} = 17, p < 0.001$	lsmeans (Bonferroni correction):<br>GF vs 0.25 FOL: $p < 0.001$<br>GF vs 0.5 FOL: $p < 0.001$<br>GF vs 1.25 FOL: $p < 0.001$<br>All other comparisons: $p > 0.05$

Analysis	Response variable	Predictor	Random effect	Sample size	Test result	Comparisons
Glmm (binomial)	% adults	Folate concentration	Replicate	GF (n=19); 0.25 FOL (n=19); 0.5 FOL (n=19); 1.25 FOL (n=20)	$F_{3,52}$ = 17, p < 0.001	lsmeans (Bonferroni correction):<br>GF vs 0.25 FOL: p < 0.001<br>GF vs 0.5 FOL: p < 0.001<br>GF vs 1.25 FOL: p < 0.001<br>All other comparisons: p > 0.05
**Figure S11. Duration of development with folate**						
Glmm	Time to pupa	Folate concentration	Replicate	Replicate A:GF (n=48); 0.25 FOL (n=48); 0.5 FOL (n=46); 1.25 FOL (n=48)	$F_{3,151}$ = 1.4, p = 0.26	/
Glmm	Time (L3)	Folate concentration	Replicate	Replicate B: GF (n=42); 0.25 FOL (n=24); 0.5 FOL (n=24); 1.25 FOL (n=24)	$F_{3,376}$ = 0.68, p = 0.57	/
Glmm	Time (L4)	Folate concentration	Replicate	Replicate C: GF (n=19); 0.25 FOL (n=19); 0.5 FOL (n=19); 1.25 FOL (n=20)	$F_{3,162}$ = 1.4, p = 0.26	/
Glmm	Time (pupal stage)	Folate concentration	Replicate		$F_{3,152}$ = 1.1, p = 0.34	/
**Figure S12. Effect of folate on L1 axenic larvae**						
				WT (n=48); GF+FOL (n=24)		
**Figure 6d. Gut lipid quantification**						
Glmm	Fluorescence/Area	Rearing condition	Replicate	Replicate A: GF (n=12); WT(n=9); WT transf (n=14)<br>Replicate B: GF (n=19); WT(n=18); WT transf (n=13)<br>Replicate C: GF (n=13); WT(n=16); WT transf (n=15)	$F_{2,124}$ = 7.7, p < 0.001	lsmeans (Bonferroni correction):<br>GF vs WT: p = 0.0026<br>GF vs WT transf: p = 0.0033<br>WT vs WT transf: p = 1
**Figure 6e. Pelt lipid quantification**						
Glmm	Fluorescence	Rearing condition	Replicate	Replicate A: GF (n=12); WT(n=12); WT transf (n=12)<br>Replicate B: GF (n=12); WT(n=12); WT transf (n=12)<br>Replicate C: GF (n=10); WT(n=10); WT transf (n=10)	$F_{2,97}$ = 18, p < 0.001	lsmeans (Bonferroni correction):<br>GF vs WT: p < 0.001<br>GF vs WT transf: p = 0.0045<br>WT vs WT transf: p = 0.019
**Figure 6f. Pelt DNA quantification**						
Glmm	Fluorescence	Rearing condition	Replicate	Replicate A: GF (n=12); WT(n=12); WT transf (n=12)<br>Replicate B: GF (n=12); WT(n=12); WT transf (n=12)<br>Replicate C: GF (n=10); WT(n=10); WT transf (n=10)	$F_{2,97}$ = 16, p < 0.001	lsmeans (Bonferroni correction):<br>GF vs WT: p < 0.001<br>GF vs WT transf: p = 0.0019<br>WT vs WT transf: p = 0.12
**Figure 6g. Larval length**						
Glmm	Length	Rearing condition	Replicate	Replicate A: GF (n=10); WT(n=9); WT transf (n=9)<br>Replicate B: GF (n=24); WT(n=24); WT transf (n=24)<br>Replicate C: GF (n=12); WT(n=6); WT transf (n=12)	$F_{2,125}$ = 18, p < 0.001	lsmeans (Bonferroni correction):<br>GF vs WT: p = 0.001<br>GF vs WT transf: p < 0.001<br>WT vs WT transf: p = 0.77
**Figure S10b. Hypoxia measurements**						
Glmm	Fluorescence	Rearing condition	Replicate	Replicate A: GF (n=10); WT(n=10); WT transf (n=10)<br>Replicate B: GF (n=10); WT(n=10); WT transf (n=10)<br>Replicate C: GF (n=9); WT(n=9); WT transf (n=9)	$F_{2,82}$ = 0.52, p = 0.60	/
**Figure S13. qPCR**						

Analysis	Response variable	Predictor	Random effect	Sample size	Test result	Comparisons
Glmm	Log$_2$ Ratio *Hexamerin 2 beta* (*AAEL011169*, *AAEL013757*)	Rearing condition	Replicate	Replicate A:<br>GF (n=1, pool of 24); GF+FOL (n=1, pool of 24);<br>WT (n=1, pool of 18); WT+FOL (n=1, pool of 18);<br>WT transf (n=1, pool of 24); WT transf + FOL (n=1, pool of 24)<br><br>Replicate B:<br>GF (n=1, pool of 24); GF+FOL (n=1, pool of 24);<br>WT (n=1, pool of 22); WT+FOL (n=1, pool of 22);<br>WT transf (n=1, pool of 24); WT transf + FOL (n=1, pool of 24)<br><br>Replicate C:<br>GF (n=1, pool of 24); GF+FOL (n=1, pool of 24);<br>WT (n=1, pool of 24); WT+FOL (n=1, pool of 24);<br>WT transf (n=0); WT transf + FOL (n=0)	$F_{3,66} = 14$, $p < 0.001$	lsmeans (Bonferroni correction):<br>WT vs WT-FOL: $p = 1$<br>GF vs GF-FOL: $p = 1$<br>GF vs WT: $p < 0.001$
Glmm	Log$_2$ Ratio *Hexamerin 2 beta* (*AAEL008817*)	Rearing condition	Replicate		$F_{3,66} = 3.4$, $p = 0.023$	lsmeans (Bonferroni correction):<br>WT vs WT-FOL: $p = 1$<br>GF vs GF-FOL: $p = 1$<br>GF vs WT: $p = 0.23$
Glmm	Log$_2$ Ratio *Hexamerin 2 beta* (*AAEL013981*, *AAEL013983*)	Rearing condition	Replicate		$F_{3,66} = 14$, $p < 0.001$	lsmeans (Bonferroni correction):<br>WT vs WT-FOL: $p = 1$<br>GF vs GF-FOL: $p = 1$<br>GF vs WT: $p < 0.001$
Glmm	Log$_2$ Ratio *Hexamerin 2 beta* (*AAEL008045*)	Rearing condition	Replicate		$F_{3,66} = 7.2$, $p < 0.001$	lsmeans (Bonferroni correction):<br>WT vs WT-FOL: $p = 1$<br>GF vs GF-FOL: $p = 1$<br>GF vs WT: $p = 0.0097$
Glmm	Log$_2$ Ratio *Alkaline phosphatase* (*AAEL000931*)	Rearing condition	Replicate		$F_{3,66} = 6.5$, $p < 0.001$	lsmeans (Bonferroni correction):<br>WT vs WT-FOL: $p = 1$<br>GF vs GF-FOL: $p = 1$<br>GF vs WT: $p = 0.006$
Glmm	Log$_2$ Ratio *Alkaline phosphatase* (*AAEL003317*)	Rearing condition	Replicate		$F_{3,66} = 1.2$, $p = 0.32$	/
Glmm	Log$_2$ Ratio *Gamma-glutamyl hydrolase* (*AAEL000271*)	Rearing condition	Replicate		$F_{3,66} = 0.52$, $p = 0.67$	/
Glmm	Log$_2$ Ratio *Lipophorin* (*AAEL009955*)	Rearing condition	Replicate		$F_{3,66} = 2.5$, $p = 0.066$	/
Glmm	Log$_2$ Ratio *Vitellogenin*-related gene (*AAEL008598*)	Rearing condition	Replicate		$F_{3,66} = 1.7$, $p = 0.16$	/
Glmm	Log$_2$ Ratio *Vitellogenin* (*AAEL010434*)	Rearing condition	Replicate		$F_{3,66} = 1.4$, $p = 0.25$	/

For each figure, the type of analysis, response variable (dependent variable), predictor (independent variable), random effect, sample size in each replicate, test results and multiple comparisons (if applicable) are shown. Glmm: generalised linear mixed model. lsmeans: least square means.

Table S2. Number of up- and down-regulated genes in germ-free larvae calculated by DESeq2.

Number of up- and down-regulated genes in germ-free larvae							
		**Gut 12h**	**Gut 20h**	**Whole larvae 12h**	**Whole larvae 20h**	**Common**	**Total unique genes**
Up-regulated genes in germ-free larvae	*padj* < 0.001	372	614	441	976	121	1919
	padj < 0.01	581	910	671	1312	184	2738
	padj < 0.05	864	1304	929	1737	269	3758
Down-regulated genes in germ-free larvae	*padj* < 0.001	295	579	270	580	67	1456
	padj < 0.01	493	859	520	966	141	2274
	padj < 0.05	766	1276	881	1475	240	3438
Number of up- and down-regulated genes in germ-free larvae with $\log_2$ fold values < -1.5 or > 1.5.							
		**Gut 12h**	**Gut 20h**	**Whole larvae 12h**	**Whole larvae 20h**	**Common**	**Total unique genes**
Up-regulated genes in germ-free larvae	*padj* < 0.001	100	203	114	380	25	697
	padj < 0.01	113	246	134	414	26	803
	padj < 0.05	130	289	154	472	28	933
Down-regulated genes in germ-free larvae	*padj* < 0.001	99	107	44	138	8	356
	padj < 0.01	131	125	62	167	10	445
	padj < 0.05	161	151	85	191	12	540

Upper section: Number of up- and down-regulated genes in germ-free larvae. Lower section: Number of up- and down-regulated genes in germ-free larvae with $\log_2$ fold values < -1.5 or > 1.5. In each table, results are shown with several statistical thresholds. *padj* is the adjusted *p* value calculated by DESeq2. "Common" refers to genes up- or down-regulated in all sample types in the same gnotobiotic condition. "Total unique genes" refers to the total number of genes up- or -down-regulated in at least one sample type. Source Data are provided as a Source Data file.

Table S3. Gene Ontology (GO) and KEGG terms significantly enriched in up-regulated (red) and down-regulated (blue) genes in germ-free larvae compared to colonised larvae.

Sample	Source	Term name	Term ID	Adjusted *p* value	n
Gut, 12H	GO:MF	oxidoreductase activity, acting on paired donors, with incorporation or reduction of molecular oxygen	GO:0016705	8.5E-07	12
	GO:MF	heme binding	GO:0020037	8.9E-07	12
	GO:MF	tetrapyrrole binding	GO:0046906	9.4E-07	12
	GO:MF	monooxygenase activity	GO:0004497	1.6E-06	11
	GO:MF	cofactor binding	GO:0048037	1.1E-05	15
	GO:MF	iron ion binding	GO:0005506	1.3E-05	11
	GO:MF	alkaline phosphatase activity	GO:0004035	2.1E-05	4
	GO:MF	catalytic activity	GO:0003824	4.5E-05	42
	GO:MF	oxidoreductase activity	GO:0016491	2.1E-03	15
	GO:MF	phosphatase activity	GO:0016791	6.4E-03	6
	GO:MF	phosphoric ester hydrolase activity	GO:0042578	3.0E-02	6
	GO:BP	oxidation-reduction process	GO:0055114	1.5E-02	16
	KEGG	Folate biosynthesis	KEGG:00790	1.9E-03	3
	KEGG	Thiamine metabolism	KEGG:00730	7.3E-03	2
	GO:MF	phospholipase A1 activity	GO:0008970	3.6E-02	2
Gut, 20H	GO:MF	alkaline phosphatase activity	GO:0004035	2.1E-04	4
	GO:MF	cofactor binding	GO:0048037	3.8E-03	16
	GO:MF	oxidoreductase activity, acting on paired donors, with incorporation or reduction of molecular oxygen	GO:0016705	1.5E-02	10
	GO:MF	heme binding	GO:0020037	1.6E-02	10
	GO:MF	tetrapyrrole binding	GO:0046906	1.6E-02	10
	GO:MF	iron ion binding	GO:0005506	1.9E-02	10
	GO:MF	catalytic activity	GO:0003824	2.0E-02	58
	GO:MF	monooxygenase activity	GO:0004497	2.0E-02	9

	**GO:MF**	metallocarboxypeptidase activity	GO:0004181	3.4E-02	4
	**GO:CC**	extracellular region	GO:0005576	8.4E-04	12
	**KEGG**	Steroid biosynthesis	KEGG:00100	1.6E-02	2
	**KEGG**	Thiamine metabolism	KEGG:00730	3.9E-02	2
Whole larvae, 12h	**GO:MF**	catalytic activity	GO:0003824	1.3E-05	46
	**GO:MF**	alkaline phosphatase activity	GO:0004035	4.1E-05	4
	**GO:MF**	heme binding	GO:0020037	3.2E-04	10
	**GO:MF**	tetrapyrrole binding	GO:0046906	3.4E-04	10
	**GO:MF**	oxidoreductase activity, acting on paired donors, with incorporation or reduction of molecular oxygen	GO:0016705	2.6E-03	9
	**GO:MF**	iron ion binding	GO:0005506	3.2E-03	9
	**GO:MF**	monooxygenase activity	GO:0004497	5.4E-03	8
	**GO:MF**	phosphatase activity	GO:0016791	1.4E-02	6
	**GO:MF**	structural constituent of cuticle	GO:0042302	4.0E-02	8
	**GO:MF**	cofactor binding	GO:0048037	4.3E-02	11
	**KEGG**	Thiamine metabolism	KEGG:00730	5.0E-04	2
	**KEGG**	Folate biosynthesis	KEGG:00790	5.7E-03	2
	**GO:MF**	structural constituent of cuticle	GO:0042302	7.6E-04	7
	**GO:MF**	structural molecule activity	GO:0005198	3.0E-02	7
	**KEGG**	Steroid biosynthesis	KEGG:00100	5.0E-02	1
Whole larvae, 20h	**GO:MF**	structural constituent of cuticle	GO:0042302	4.8E-08	21
	**GO:MF**	structural molecule activity	GO:0005198	9.5E-04	21

**GO:MF**	chitin binding	GO:0008061	1.1E-03	12
**GO:MF**	alkaline phosphatase activity	GO:0004035	1.4E-03	4
**GO:MF**	endopeptidase activity	GO:0004175	3.0E-02	20
**GO:MF**	peptidase activity, acting on L-amino acid peptides	GO:0070011	4.4E-02	24
**GO:BP**	chitin metabolic process	GO:0006030	4.6E-05	12
**GO:BP**	glucosamine-containing compound metabolic process	GO:1901071	5.5E-05	12
**GO:BP**	amino sugar metabolic process	GO:0006040	6.4E-05	12
**GO:BP**	aminoglycan metabolic process	GO:0006022	1.4E-04	12
**GO:BP**	metabolic process	GO:0008152	2.1E-04	79
**GO:BP**	proteolysis	GO:0006508	9.0E-04	27
**GO:BP**	drug metabolic process	GO:0017144	5.1E-03	12
**GO:CC**	extracellular region	GO:0005576	2.1E-07	20
**KEGG**	Thiamine metabolism	KEGG:00730	9.8E-03	2
**GO:MF**	serine-type peptidase activity	GO:0008236	2.2E-03	12
**GO:MF**	serine hydrolase activity	GO:0017171	2.2E-03	12
**GO:MF**	serine-type endopeptidase activity	GO:0004252	6.4E-03	11
**GO:MF**	oxidoreductase activity, acting on the aldehyde or oxo group of donors, NAD or NADP as acceptor	GO:0016620	3.9E-02	4
**KEGG**	Tyrosine metabolism	KEGG:00350	3.7E-02	2
**KEGG**	Metabolic pathways	KEGG:01100	4.0E-02	7

The analysis was performed in g:Profiler. Statistical significance was assessed with a Fisher's one tail test with Bonferroni correction. n indicates the number of genes associated with that term that are significantly regulated in the indicated sample. GO:MF: Gene Ontology, Molecular Function; GO:BP: Gene Ontology, Biological Process; GO:CC: Gene Ontology, Cell Compartment. Source Data are provided as a Source Data file.

Table S4. List of the most up-regulated genes in germ-free larvae compared to colonised larvae.

AAEL	Description	Phosphatase	Hydrolytic reaction	Chitin binding	Transferase activity	Immunity	Cuticle constituent	Lipid metabolism	Other functions	germ-free vs *E. coli*			
										GUT_12h	GUT_20h	WL_12h	WL_20h
000931	Alkaline phosphatase	■								9.2	7.2	5.5	5.6
003317	Alkaline phosphatase	■								7.1	6.1	7.6	6.0
003297	Alkaline phosphatase	■								5.8	3.2	5.4	3.2
002555	Sodium/solute symporter								Transmembrane transport	5.7	4.5	3.6	3.1
002138	Triacylglycerol lipase		■					■		4.4	3.9	4.0	4.0
003289	Alkaline phosphatase	■								5.2	3.0	4.7	3.1
000323	Cysteine-rich venom protein								Trypsine inhibitor activity	4.0	3.9	3.7	3.6
029062	Unknown (Chitin binding)			■						5.0	3.0	4.5	2.7
024870	Nose resistant to fluoxetine protein 6-like				■					3.3	3.1	3.8	3.1
003905	Alkaline phosphatase	■								4.5	2.5	3.1	3.1
012636	Cytochrome b5								Oxidoreductase activity	3.0	3.5	3.0	2.7
014246	Glucosyl/glucuronosyl transferases				■					4.1	2.7	3.7	2.6
017359	Unknown									-1.3	5.8	-	4.4
012955	Phosphatidylethanolamine-binding protein							■		3.4	3.7	2.0	1.1
023929	Unknown									-	4.5	1.1	4.4

AAEL	Description	Phosphatase	Hydrolytic reaction	Chitin binding	Transferase activity	Immunity	Cuticle constituent	Lipid metabolism	Other functions	germ-free vs *E. coli*			
										GUT_12h	GUT_20h	WL_12h	WL_20h
001323	Protein takeout								Odorant binding activity	-	-	8.4	-
003358	Unknown									-	3.8	-	4.3
012737	Nose resistant to fluoxetine protein 6									1.6	4.2	1.6	3.9
012164	SPZ6: spaetzle-like cytokine									-	3.2	0.5	4.8
027145	Unknown (Serpin)									-	4.2	0.7	3.6
012642	Unknown (Chitin binding)									-	3.4	0.3	4.3
001498	Unknown									-	3.4	0.5	4.1
025451	Angiopoietin-related protein 2									-	-	-	7.0
015559	Zinc carboxypeptidase								Hydrolytic reaction	-	2.5	-	4.5
025440	lncRNA									-	-	3.7	2.9
024040	ATP synthase subunit b, mitochondrial-like								Proton-transporting ATP synthase complex	-	6.4	-	-
002961	Osiris, putative									-	-	0.9	6.3
003242	Pupal cuticle protein									6.3	-	-	-1.4
003351	Unknown									-	-	1.0	6.0
005024	Unknown									-	-	1.1	4.6
010631	Unknown (Orthologue to Osiris)									-	-	-	5.4
010628	Unknown (Orthologue to Osiris)									-	-	-	5.1
027763	Lactoylglutathione lyase									-	-	-	4.9

AAEL	Description	Phosphatase	Hydrolytic reaction	Chitin binding	Transferase activity	Immunity	Cuticle constituent	Lipid metabolism	Other functions	germ-free vs *E. coli*			
										GUT_12h	GUT_20h	WL_12h	WL_20h
025469	Preli-like protein									-	-	4.9	-
023249	Unknown									-	-	-	4.9
004030	Unknown									-	-	-	4.8
003337	Unknown									-1.1	3.4	-	4.7
014766	Nose resistant to fluoxetine protein 6									-	-	4.6	-
025898	MAGE-like protein 2									-	-	-	4.5
020544	Pseudogene									4.3	-	2.9	-
001294	Unknown (Haemolymph_juvenile_hormone-bd)									-	-	3.7	-1.7
013031	APG18A: autophagy related gene									-	-	5.2	-4.1

For each gene, Vectorbase accession, Vectorbase description, predicted function and expression values in the different samples are indicated. Different colour codes indicate genes with a shared function. Significant expression values (*p*adj < 0.001) are coloured with a blue/red colour code corresponding to down/up-regulation. Hyphens mean absence from the DESeq2 output of genes with *p*adj < 0.1, meaning that transcripts are either not detected or not differentially regulated at this 0.1 threshold. Exact *p* values and Source Data are provided as a Source Data file.

Table S5. List of the most down-regulated genes in germ-free larvae compared to colonised larvae.

AAEL	Description	Amino acid storage	Hydrolytic reaction	Lipid metabolism	Cuticle constituent	Immunity	Other functions	germ-free vs *E. coli*			
								GUT_12h	GUT_20h	WL_12h	WL_20h
008045	Hexamerin 2 beta							-	-6.7	-5.3	-7.0
011169	Hexamerin 2 beta							-3.6	-4.2	-2.5	-4.2
011520	Sucrose transport protein						Sucrose transport	-4.5	-3.8	-3.1	-2.9
008817	Hexamerin 2 beta							-	-4.0	-3.1	-6.0
018240	Unknown (Fuseless region)							-3.3	-3.4	-3.3	-3.0
008313	Unknown							-2.9	-3.1	-4.5	-4.5
013757	Hexamerin 2 beta							-	-5.4	-2.2	-4.5
011661	Unknown (Cilia structure/activity)							-3.9	-4.1	-1.9	-3.2
023132	Unknown (Lipase activity)							-2.7	-4.0	-2.5	-4.5
008598	Unknown (Vitellogenin domain)							-	-2.7	-2.0	-4.7
013981	Hexamerin 2 beta							-	-4.5	-1.7	-4.4
004341	Carboxy/choline esterase Alpha Esterase							-	-	-	-8.9
014886	4-aminobutyrate aminotransferase							-3.2	-4.1	-0.4	-1.0
011968	Unknown							-6.8	-	-	-
011349	Serine protease							-1.8	-3.5	-0.7	-1.4
022253	Pseudogene							-4.0	-	-2.5	-
006824	Cytochrome P450						Oxidoreductase activity	-	-	-	-6.4

AAEL	Description	Amino acid storage	Hydrolytic reaction	Lipid metabolism	Cuticle constituent	Immunity	Other functions	germ-free vs *E. coli*			
								GUT_12h	GUT_20h	WL_12h	WL_20h
012311	Vitellogenin			X				-	-3.8	-	-2.3
002237	Fatty acid synthase			X				-	-	-	-6.1
004343	OBP19: odorant bp							-	-1.7	-2.7	-3.3
014994	Unknown (Cuticular protein)				X		Cuticle constituent	-6.7	-	-	1.0
024321	SWI/SNF complex subunit SMARCC2-like						DNA binding	-	-5.5	-	-
011407	CTL20: C-Type Lectin					X		-2.2	-1.8	-3.1	-1.9
003890	Cytochrome P450							-	-	-3.2	-1.9
017976	HSP70Bb: heat shock protein							-3.6	-	-1.5	-
024362	Tetra-peptide repeat homeobox protein 1-like						DNA binding	-6.4	-	-1.6	1.6
013983	Hexamerin 2 beta	X						-	-4.5	-2.2	-4.7
011944	CCEAE3O: carboxy/choline esterase Alpha Esterase		X					-	-	-	-4.6
025320	Cyclin-dependent kinase inhibitor 1C-like						Cell cycle regulator	-4.5	1.3	-	0.8
000689	Steroid dehydrogenase							-	-	-	-4.2
013031	Unknown (Cuticular protein)				X			-	-	5.2	-4.1
028198	Carboxylic ester hydrolase (Fragment)		X					-4.1	-	-3.4	-
009524	MAL1: alpha-amylase		X					-	-4.1	-	-2.4
021670	Unknown (Odorant binding protein)							-	-	-3.1	-4.1

AAEL	Description	Amino acid storage	Hydrolytic reaction	Lipid metabolism	Cuticle constituent	Immunity	Other functions	germ-free vs *E. coli*			
								GUT_12h	GUT_20h	WL_12h	WL_20h
022059	Pseudogene							-3.7	-	-2.0	-
010134	Pupal cuticle protein, putative				■			-	-3.7	-	-
013152	Leucine-rich repeat extensin-like protein 3							-3.5	-	-	4.0
026388	Cuticle protein 38-like				■			-	-	-3.4	-
027210	Nuclear pore complex protein DDB_G0274915-like						Transport into nucleus	-4.9	-	-0.8	2.4
027233	Unknown							-	-	-4.3	2.7
000914	Cuticle protein				■			-	-	-5.1	-1.2
004953	Elongation of very long chain fatty acids protein			■				-	1.5	-2.8	1.9
022796	Unknown							-	-	-2.9	3.4

For each gene, Vectorbase accession, Vectorbase description, predicted function and expression values in the different samples are indicated. Different colour codes indicate genes with a shared function. Significant expression values ($p$adj < 0.001) are coloured with a blue/red colour code corresponding to down/up-regulation. Hyphens means absence from the DESeq2 output of genes with $p$adj < 0.1, meaning that transcripts are either not detected or not differentially regulated at this 0.1 threshold. Exact p values and Source Data are provided as a Source Data file.

Table S6. Expression values of genes encoding for folate transporters.

AAEL	Description	germ-free vs *E. coli*	germ-free vs *E. coli*	germ-free vs *E. coli*	germ-free vs *E. coli*
		GUT_12h	GUT_20h	WL_12h	WL_20h
001687	Proton-coupled folate transporter	2.008	2.581	1.786	2.340
001047	Proton-coupled folate transporter	1.775	1.268	0.874	0.494
001697	Proton-coupled folate transporter	-	2.236	-	-
021114	Proton-coupled folate transporter	-	-	-	-
001691	Proton-coupled folate transporter	-	-	-	-
027091	Folate transporter 1	-	-	-	-
006879	Folate carrier protein	-	-0.360	-	-
003983	Proton-coupled folate transporter	-	-	0.782	-1.064
009513	Proton-coupled folate transporter	-	-	-	-1.988

For each gene, Vectorbase accession (AAEL), Vectorbase description, and expression values in the different samples are indicated. Significant expression values ($p$adj < 0.001) are coloured with a blue/red colour code corresponding to down/up-regulation. Hyphens mean absence from the DESeq2 output of genes with $p$adj < 0.1, meaning that transcripts are either not detected or not differentially regulated at this 0.1 threshold. Exact p values and Source Data are provided as a Source Data file.

Table S7. Primer sequences used in this study.

Gene name or description	Vectorbase accession	Forward primer	Reverse primer	Reference
16S	-	TCCTACGGGAGGCAGCAGT	GGACTACCAGGGTATCTAATCCTGTT	[2]
S17	AAEL004175	AAGAAGTGGCCATCATTCCA	GGTCTCCGGGTCGACTTC	[3]
Vitellogenin	AAEL010434	CTTCTCGCTTTGGCGGGG	CCTGGTAGGCGTTCTGATATCC	-
Hexamerin 2 beta	AAEL008045	TCCAAGATGCTGCTCAGTGG	ACGACACCCTTGAAGCTGAG	-
Hexamerin 2 beta	AAEL008817	GGTGATCCCAAGTGTCCTGG	CCGGTCGAAGTACGTCACAA	-
Hexamerin 2 beta	AAEL011169 AAEL013757	CGTACTACTACTACTTCCACGCTG	TCGTTGGACAAGCGTTCCA	-
Hexamerin 2 beta	AAEL013981 AAEL013983	CTCGTCAGAAGCGAATCAACC	CGACGAAGTCATACACGTATTGATC	-
Unknown (Vitellogenin-related gene)	AAEL008598	ACAATTGGGCCGTCTACGTT	CCCGAGACAACTCCATGCTT	-
Lipophorin	AAEL009955	GTGGATACCGCGAGTCTCTG	CGTCAGCAGTGGAGTGGATT	-
Alkaline phosphatase	AAEL000931	TCGGTTACGCTAATCGACCG	GTAGAAGGCCGATAGGTGCC	-
Alkaline phosphatase	AAEL003317	ATGCAGTTACCGGGGATGTC	TTGGTTGGAACCTCGGATGG	-
Gamma-glutamyl hydrolase	AAEL000271	ATTGAGACGGCCAAAGGTCC	TCGTTGGCGCATTGAACTTG	-

For each gene, the Vectorbase accession (if applicable), 5'-3' sequence of the forward and reverse primers and references (if applicable) are indicated.

Supplementary references

1. Cuenca, M. *et al.* D-Alanine-controlled transient intestinal mono-colonization with non-laboratory-adapted commensal *E. coli* strain HS. *PLoS One* **11**, e0151872 (2016).

2. Nadkarni, M. A., Martin, F. E., Jacques, N. A. & Hunter, N. Determination of bacterial load by real-time PCR using a broad-range (universal) probe and primers set. *Microbiology* **148**, 257–66 (2002).

3. Dzaki, N., Ramli, K. N., Azlan, A., Ishak, I. H., Azzam, G. Evaluation of reference genes at different developmental stages for quantitative real-time PCR in *Aedes aegypti*. *Scientific Reports* **7**, 43618 (2017).
