# Supplementary material for: Production of germ-free mosquitoes via transient colonisation allows stage-specific investigation of host–microbiota interactions
Source: Nat Commun. 2021 Feb 11;12:942. doi: 10.1038/s41467-021-21195-3 (PMC7878806; doi:10.1038/s41467-021-21195-3)
Supplement: Supplementary file 3 — Reporting Summary [file 41467_2021_21195_MOESM3_ESM.pdf]

nature research

Corresponding author(s): Mathilde Gendrin, Ottavia Romoli

Last updated by author(s): Jan 8, 2021

Reporting Summary

Nature Research wishes to improve the reproducibility of the work that we publish. This form provides structure for consistency and transparency in reporting. For further information on Nature Research policies, see our Editorial Policies and the Editorial Policy Checklist.

Statistics

For all statistical analyses, confirm that the following items are present in the figure legend, table legend, main text, or Methods section.

n/a	Confirmed	
☐	☒	The exact sample size (*n*) for each experimental group/condition, given as a discrete number and unit of measurement
☐	☒	A statement on whether measurements were taken from distinct samples or whether the same sample was measured repeatedly
☐	☒	The statistical test(s) used AND whether they are one- or two-sided<br>*Only common tests should be described solely by name; describe more complex techniques in the Methods section.*
☒	☐	A description of all covariates tested
☐	☒	A description of any assumptions or corrections, such as tests of normality and adjustment for multiple comparisons
☐	☒	A full description of the statistical parameters including central tendency (e.g. means) or other basic estimates (e.g. regression coefficient) AND variation (e.g. standard deviation) or associated estimates of uncertainty (e.g. confidence intervals)
☐	☒	For null hypothesis testing, the test statistic (e.g. *F*, *t*, *r*) with confidence intervals, effect sizes, degrees of freedom and *P* value noted<br>*Give P values as exact values whenever suitable.*
☒	☐	For Bayesian analysis, information on the choice of priors and Markov chain Monte Carlo settings
☒	☐	For hierarchical and complex designs, identification of the appropriate level for tests and full reporting of outcomes
☒	☐	Estimates of effect sizes (e.g. Cohen's *d*, Pearson's *r*), indicating how they were calculated

Our web collection on statistics for biologists contains articles on many of the points above.

Software and code

Policy information about availability of computer code

Data collection	Image Focus (Euromex, imaging with stereomicroscope), EVOS FL Auto (Life Technologies, fluorescence microscopy imaging), Light Cycler 480 (Roche, qPCR), NextSeq 500 sequencing platform (Illumina).
Data analysis	GraphPad Prism (v 8.4.3), R (v 3.6.0), Excel for Mac (v 16.16.27), FIJI (v 2.0.0), FastQC (v 0.11.9), Trimmomatic (v 0.39), HISAT2 (v 2.1.0), featureCounts (v 1.6.4), g::Profiler, DESeq2 (v 1.24.0)

For manuscripts utilizing custom algorithms or software that are central to the research but not yet described in published literature, software must be made available to editors and reviewers. We strongly encourage code deposition in a community repository (e.g. GitHub). See the Nature Research guidelines for submitting code & software for further information.

Data

Policy information about availability of data

All manuscripts must include a data availability statement. This statement should provide the following information, where applicable:

- Accession codes, unique identifiers, or web links for publicly available datasets
- A list of figures that have associated raw data
- A description of any restrictions on data availability

All data generated during this study are available as a Source Data file. The raw RNA sequencing data underlying Figure 5 are available in the Sequencing Read Archive (SRA) under the Bioproject ID PRJNA687261 . Mosquito cartoons are available under a CC-BY-NC-SA 4.0 license on our laboratory website (https://microbiota-insect-vectors.group/en/diy). Materials and additional data are available from corresponding authors upon request.

Field-specific reporting

Please select the one below that is the best fit for your research. If you are not sure, read the appropriate sections before making your selection.

☒ Life sciences ☐ Behavioural & social sciences ☐ Ecological, evolutionary & environmental sciences

For a reference copy of the document with all sections, see nature.com/documents/nr-reporting-summary-flat.pdf

Life sciences study design

All studies must disclose on these points even when the disclosure is negative.

Sample size

Sample sizes were chosen based on previously published litterature for the performed assays:
- larval development: (1); (2)
- CFU measurements: (2)
- qPCR experiments on bacterial DNA: (3)
- larval length: (2)
- wing length: (3)
- reproduction success: (3)
- lifespan: (3)
- transcriptome sequencing: (2). We aimed for 60 individuals because MG's previous work on Drosophila larvae (4) based on 30 individuals/samples was not precise enough to recover a perfect clusterisation of samples by replicate while another study based on 60 Drosophila guts/sample, did (5).The sample distance matrix confirmed the clusterisation of all the samples by replicate, validating the chosen sample size.
- hypoxia measurements: (6)
- lipid quantification: (7)
- qPCR experiments on gene expression: (8)

(1) Coon, K. L., Vogel, K. J., Brown, M. R. & Strand, M. R. Mosquitoes rely on their gut microbiota for development. Mol. Ecol. 23, 2727–2739 (2014).
(2) Vogel, K. J., Valzania, L., Coon, K. L., Brown, M. R. & Strand, M. R. Transcriptome sequencing reveals large-scale changes in axenic Aedes aegypti larvae. PLoS Negl. Trop. Dis. 11, e0005273 (2017).
(3) Correa, M. A., Matusovsky, B., Brackney, D. E. & Steven, B. Generation of axenic Aedes aegypti demonstrate live bacteria are not required for mosquito development. Nat. Commun. 9, 4464 (2018).
(4) Gendrin, M., Zaidman-Rémy, A., Broderick, N.A., Paredes, J., Poidevin, M., Roussel, A., Lemaitre, B. Functional Analysis of PGRP-LA in Drosophila Immunity. PLoS ONE 8(7): e69742 (2013).
(5) Buchon N, Broderick NA, Poidevin M, Pradervand S, Lemaitre B. Drosophila intestinal response to bacterial infection: activation of host defense and stem cell proliferation. Cell Host Microbe. Feb 19;5(2):200-11. doi: 10.1016/j.chom.2009.01.003. PMID: 19218090 (2009).
(6) Coon, K. L. et al. Bacteria-mediated hypoxia functions as a signal for mosquito development. Proc. Natl. Acad. Sci. U.S.A 114, E5362–E5369 (2017).
(7) Valzania, L., Coon, K. L., Vogel, K. J., Brown, M. R. & Strand, M. R. Hypoxia-induced transcription factor signaling is essential for larval growth of the mosquito Aedes aegypti. Proc. Natl. Acad. Sci. U.S.A 115, 457–465 (2018).
(8) Roy, S., Saha, T. T., Johnson, L., Zhao, B., Ha, J., White, K. P., Girke, T., Zou, Z., & Raikhel, A. S. Regulation of Gene Expression Patterns in Mosquito Reproduction. PLoS genetics, 11(8), e1005450 (2015). https://doi.org/10.1371/journal.pgen.1005450

Data exclusions

With some egg batches we observed that all larvae completed their development upon transfer despite their rearing condition. We discarded those data and we considered valid and experiment when WT E. coli gnotobiotic larvae transferred to new rearing medium had a 40-60 % developmental success. This is specified in the materials and methods section. When blood-fed mosquitoes were used, non blood-fed or partially blood-fed mosquitoes were discarded prior to dissection or to fecundity analysis.

Replication

At least three replicates on independent egg-batches were repeated for each experiment, except for Figure 4a (2 replicates), Figure S1 (2 replicates), Figure S12 (1 replicate) and Figure S13 (2 replicates for WT transferred individuals). All results were succefully replicated except for the development success of larvae upon transfer, which varied with the egg-batch analysed, as stated above.

Randomization

For transcriptome sequencing, sample processing order was inverted between replicates.
Larvae from the same egg batch were randomly allocated into the experimental groups.

Blinding

Investigators were not blinded because quantification involved either automatic analysis (qPCR, transcriptomic analysis, fluorescence quantification) or was not subject to investogator bias (CFU counts, lifespan, egg-laying, development). Moreover, the experimental setup did not allowed to complete blind operators (amino acids need to be added only to AUXO-carrying individuals) and we considered that the increase in experimental complexity linked to blinding was not useful.

Reporting for specific materials, systems and methods

We require information from authors about some types of materials, experimental systems and methods used in many studies. Here, indicate whether each material, system or method listed is relevant to your study. If you are not sure if a list item applies to your research, read the appropriate section before selecting a response.

Materials & experimental systems

n/a	Involved in the study
☒	☐ Antibodies
☒	☐ Eukaryotic cell lines
☒	☐ Palaeontology and archaeology
☐	☒ Animals and other organisms
☒	☐ Human research participants
☒	☐ Clinical data
☒	☐ Dual use research of concern

Methods

n/a	Involved in the study
☒	☐ ChIP-seq
☒	☐ Flow cytometry
☒	☐ MRI-based neuroimaging

Animals and other organisms

Policy information about studies involving animals; ARRIVE guidelines recommended for reporting animal research

Laboratory animals	Aedes aegypti New Orleans strain
Wild animals	The study did not involve wild animals
Field-collected samples	Water collected from Ae. aegypti larval breeding site was transported to the lab in autoclaved or clean glass containers. It was either immediately used to colonise germ-free larvae, or stored at room temperature on a 12:12 h light/dark cycle and used the following day. After use, it was decontaminated with bleach (1% chlorine concentration)
Ethics oversight	No protected animal was used for the experiments described in this paper. For the maintenance of the mosquito colony, mosquitoes were blood-fed on mice. Protocols have been validated by the French Direction générale de la recherche et de l'innovation, ethical board # 089, under the agreement # 973021.

Note that full information on the approval of the study protocol must also be provided in the manuscript.
